# Two Trk/Ktr/HKT-type potassium transporters, TrkG and TrkH, perform distinct functions in *Escherichia coli* K-12

**DOI:** 10.1016/j.jbc.2022.102846

**Published:** 2022-12-29

**Authors:** Ellen Tanudjaja, Naomi Hoshi, Kaneyoshi Yamamoto, Kunio Ihara, Tadaomi Furuta, Masaru Tsujii, Yasuhiro Ishimaru, Nobuyuki Uozumi

**Affiliations:** 1 Department of Biomolecular Engineering, Graduate School of Engineering, Tohoku University, Sendai, Japan; 2 Department of Frontier Bioscience, Hosei University, Tokyo, Japan; 3 Center for Gene Research, Nagoya University, Nagoya, Japan; 4 School of Life Science and Technology, Tokyo Institute of Technology, Yokohama, Japan

**Keywords:** Trk/Ktr/HKT, potassium transport, gene transfer, *Escherichia coli*, membrane protein, molecular evolution, H-NS, histone-like nucleoid structuring protein, K+, potassium ion, qRT–PCR, quantitative RT–PCR

## Abstract

*Escherichia coli* K-12 possesses two versions of Trk/Ktr/HKT-type potassium ion (K^+^) transporters, TrkG and TrkH. The current paradigm is that TrkG and TrkH have largely identical characteristics, and little information is available regarding their functional differences. Here, we show using cation uptake experiments with K^+^ transporter knockout mutants that TrkG and TrkH have distinct ion transport activities and physiological roles. K^+^-transport by TrkG required Na^+^, whereas TrkH-mediated K^+^ uptake was not affected by Na^+^. An aspartic acid located five residues away from a critical glycine in the third pore-forming region might be involved in regulation of Na^+^-dependent activation of TrkG. In addition, we found that TrkG but not TrkH had Na^+^ uptake activity. Our analysis of K^+^ transport mutants revealed that TrkH supported cell growth more than TrkG; however, TrkG was able to complement loss of TrkH-mediated K^+^ uptake in *E. coli*. Furthermore, we determined that transcription of *trkG* in *E. coli* was downregulated but not completely silenced by the xenogeneic silencing factor H-NS (histone-like nucleoid structuring protein or heat-stable nucleoid-structuring protein). Taken together, the transport function of TrkG is clearly distinct from that of TrkH, and TrkG seems to have been accepted by *E. coli* during evolution as a K^+^ uptake system that coexists with TrkH.

Potassium ion (K^+^) is an essential cation for most living organisms. It has significant roles in cytoplasmic ion homeostasis, enzymatic activation, membrane potential formation, and osmoregulation (1, 2, 3). The presence of a K^+^ uptake system in the membrane is crucial in prokaryotic cells and nonanimal eukaryotes because they lack the Na^+^/K^+^ pump present in animal cells. K^+^ uptake systems are divided into K^+^ channels and three classes of K^+^ transporters, Kdp, Kup/HAK/KT, and Trk/Ktr/HKT (4, 5). Which types and how many transporters are present varies between organisms. The *Escherichia coli* genome contains genes encoding a K^+^ channel homolog, *kch*, and all three classes of K^+^ uptake transporters, *kdp*, *kup*, and *trk*. K^+^ transport function of Kch has not been shown, but activities of the other three systems ensure that *E. coli* can satisfy its K^+^ requirements under different environmental conditions (1).

Under K^+^-limited conditions (<1 mM K^+^), the ATP-hydrolyzing K^+^ transporter, Kdp, is induced and functions as the primary K^+^ uptake route for *E. coli* survival (6). Kdp exhibits high affinity and high specificity for K^+^ (7, 8, 9). The transporter consists of the multiple subunits of the KdpFABC complex. Expression of the *kdpFABC* operon is controlled by a two-component regulatory system comprising KdpD and KdpE (10), which sense low intracellular K^+^ concentrations and high external osmolarity (6). Kdp expression is rapidly downregulated upon high K^+^ concentrations (>5 mM) (6).

When K^+^ concentrations are sufficient for the cells, Kup and Trk, which are constitutively expressed, are the main contributors to maintenance of intracellular K^+^ levels instead of Kdp. In *E. coli*, Kup has been proposed to function as a K^+^–H^+^ symporter (11, 12), forming the main K^+^ influx passage during hyperosmotic stress at low pH (13). Moreover, Kup has the ability to uptake Cs^+^ and Rb^+^, which allows *E. coli* to use Cs^+^ and/or Rb^+^ as a substitute for K^+^ under low K^+^ conditions (14, 15, 16). *E. coli* K-12 contains two Trk/Ktr/HKT homologs, TrkG and TrkH, which share 41% identity and have the same membrane topology (17, 18). Early studies have identified differences between TrkG and TrkH with regard to kinetics and SapD protein (TrkE) dependence (17, 19). In contrast, both activities depend on a cytosolic protein, TrkA, which contains an RCK domain to control K^+^ flux in *E. coli* (20, 21). The crystal structure of *Vibrio parahemolyticus* TrkH–TrkA (21, 22) reveals that a TrkH dimer forms a complex with a TrkA-tetramer ring. K^+^ transport activity of TrkH is activated by ATP binding to TrkA, which then induces conformational changes in TrkA and thereby opens the channel in TrkH (23). A similar architecture is also found in the closely related *Bacillus subtilis* KtrA–KtrB (24). Trk/Ktr/HKT transporters are present in prokaryotes as well as in eukaryotes including yeast and plants (5, 25, 26). All Trk/Ktr/HKT transporters are characterized by a common ancestral K^+^ channel structure (18, 27, 28, 29). Although they are classified as K^+^ transporters, some of them possess Na^+^-related activities, such as Na^+^-activated K^+^ transport properties (*Synechocystis* KtrABE (30) and *Vibrio alginolyticus* KtrAB (29)), K^+^–Na^+^ cotransport activity (wheat HKT1) (31), or strong Na^+^ over K^+^ selectivity for transport (*Arabidopsis thaliana* HKT1) (32). TrkG and TrkH in *E. coli* are considered ancestral Trk/Ktr/HKT-type transporters in terms of evolution, but their individual transport characteristics and potentially unique roles remain to be elucidated. Interestingly, *trkG* and *trkH* are predicted to have a different evolutionary history (33). While *trkH* is recognized to be the part of *E. coli* core genome, *trkG* is most likely a “foreign gene” that was acquired by *E. coli via* horizontal gene transfer, through bacteriophage transduction (33). This hypothesis is supported by two observations: (1) *trkG* has significantly lower GC content than *trkH* (38% *versus* 53%) and (2) *trkG* is located in the *rac* cryptic prophage. The expression of such foreign genes is usually silenced or repressed under normal conditions by H-NS (histone-like nucleoid structuring protein or heat-stable nucleoid-structuring protein), also known as xenogeneic silencing (34, 35, 36). Even though these differences are known, TrkG function has generally been extrapolated from information available for TrkH, mostly since only limited research has been done specifically on TrkG. Thus, there is a clear need to study TrkG and TrkH characteristics individually.

In this study, we characterized the ion transport properties, gene regulation, and physiological role of TrkG and TrkH in *E. coli*. TrkG exhibited Na^+^-dependent K^+^ uptake as well as Na^+^ uptake activity, whereas TrkH showed Na^+^-independent K^+^ uptake and no Na^+^ uptake activity. We confirmed that *trkG* expression was modulated by H-NS; however, this did not lead to gene silencing. Under sufficient K^+^ and Na^+^ conditions, both TrkG and TrkH functioned as important K^+^ uptake systems. These data suggest that during the course of bacterial evolution, horizontally transferred *trkG* contributed to the expansion of functional diversity in *E. coli*.

## Results

### TrkH has higher K^+^ uptake activity than TrkG

**Figure 1:**
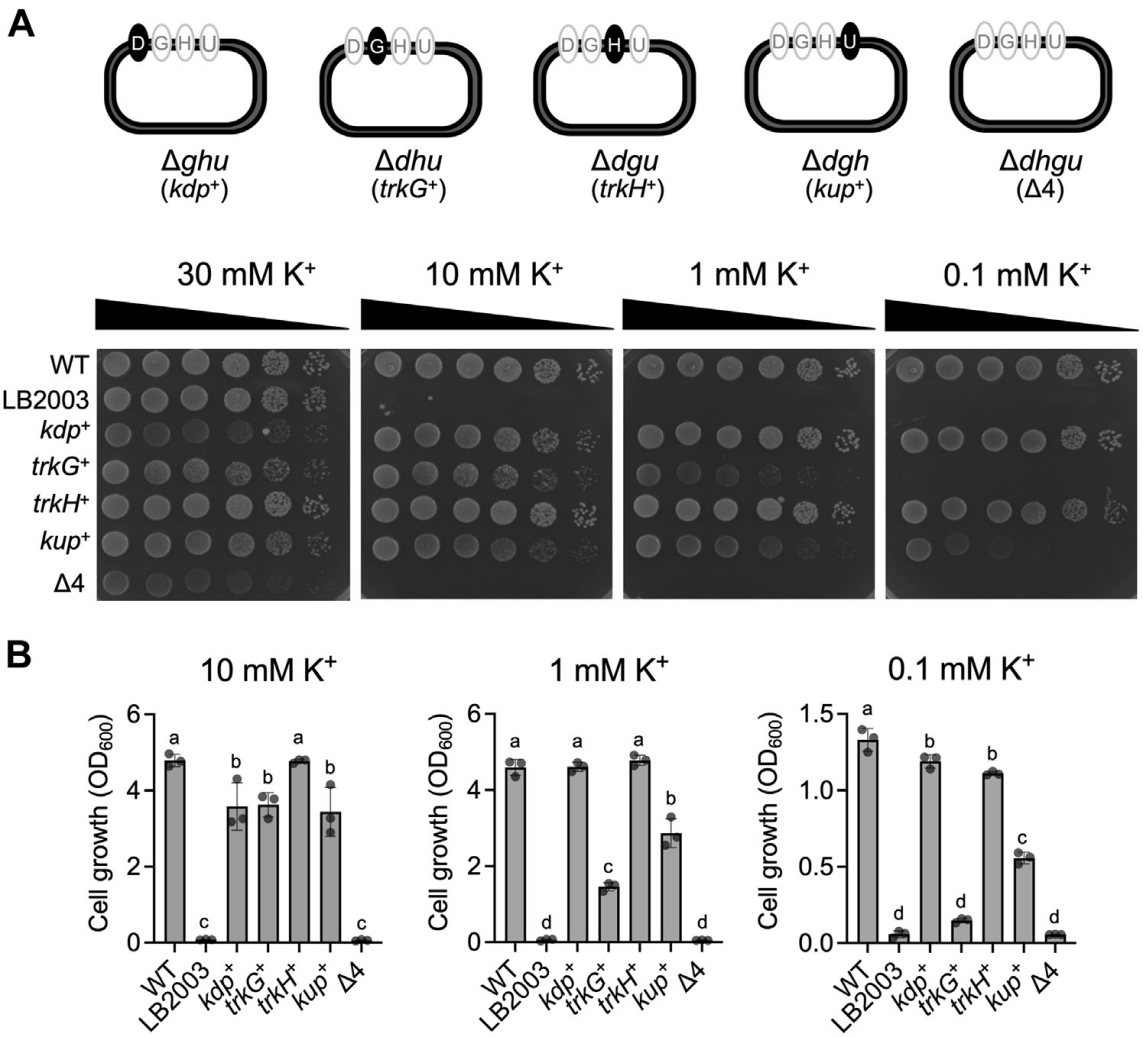
**Growth of *Escherichia coli* K**^**+**^**uptake transporter mutants in K**^**+**^**limited medium.***A*, schematic showing *E. coli* triple and quadruple knockout mutants lacking K^+^ uptake transporters in different combinations, d, *kdpA*; g, *trkG*; h, *trkH*; and u, *kup* (*top*). Growth of these mutants on K^+^-limited agar medium supplemented with different K^+^ concentrations. LB2003; Δ*ghu* (*kdp*^*+*^), Δ*dhu* (*trkG*^+^), Δ*dgu* (*trkH*^+^), Δ*dgh* (*kup*^*+*^), and Δ*dghu* (Δ4) (*lower*). Ten-fold serial dilutions of the cells were spotted on agar medium and then incubated at 30 °C for 2 days. The experiment was repeated three times; representative results are shown. *B*, growth of the same mutants in liquid culture. Cell growth was measured after overnight incubation (*t* = 15 h) at 30 °C. Mean ± SD. n = 3, biological replicates. One-way ANOVA, Tukey test, *p* < 0.05. *Different letters* above the bar in graph means statistically different. K^+^, potassium ion.

*E. coli* K-12 have two homologous Trk-type K^+^ transporters, TrkG and TrkH, whose functional properties and physiological roles have not been fully characterized (17, 33). To evaluate the functions of TrkG and TrkH in K^+^ uptake, we constructed a series of triple knockout mutants, each retaining only one of the four K^+^ transporters, Δ*dhu* (*trkG*^+^), Δ*dgu* (*trkH*^+^), Δ*ghu* (*kdp*^*+*^), and Δ*dgh* (*kup*^*+*^), as well as a quadruple knockout mutant, Δ*dghu* (Δ4). To simplify the naming of mutants, [*d*] represents *kdpA*, [*g*] represents *trkG*, [*h*] represents *trkH*, and [*u*] represents *kup*, and the alternative name in bracket describes the remaining K^+^ uptake gene in the strain. Each of the triple mutants, therefore, allowed us to assay the sole activity of one K^+^ transport system in *E. coli* (Table 1 and Fig. 1*A*, *top panel*). To compare how the K^+^ uptake ability of either TrkG or TrkH supported growth in *E. coli*, cells were grown with minimal medium containing varying concentrations of K^+^, both on plates (Fig. 1*A*, *lower panels*) or in liquid culture (Fig. 1*B*). The Δ*dgu* (*trkH*^+^) mutant, lacking *kdpA*, *trkG*, and *kup*, was able to grow well in medium with low K^+^ (0.1 mM), similar to the Δ*ghu* (*kdp*^*+*^) mutant, which still contained the ATP-driven high-affinity K^+^ uptake transporter (37). The growth of the Δ*dhu* (*trkG*^+^) and Δ*dgh* (*kup*^*+*^) strains was very low on media with 0.1 mM and 1 mM K^+^, compared with the growth of Δ*dgu* (*trkH*^+^) and Δ*ghu* (*kdp*^*+*^) (Fig. 1*B*). Furthermore, we determined growth curves for the strains under these K^+^ concentrations (Fig. S1). In comparison, the Δ*dghu* (Δ4) mutant, lacking all four major K^+^ transport systems, was only able to grow in medium supplemented with 30 mM K^+^ (Fig. 1*A*, *lower panels*). This was similar to the growth of the widely used K^+^ transport–defective *E. coli* mutant, LB2003 (38). The growth in liquid culture generally showed the same tendency as that on agar medium (Fig. 1, *A* and *B*). These results suggested that TrkH was the dominant K^+^ uptake system, and TrkG and Kup were minor contributors to K^+^ uptake in *E. coli* at 0.1 mM K^+^.

**Table 1:** Bacterial strains used in this study

Strain name	Genotype	Reference
BW25113	*F*^*−*^*Δ(araD-araB)567 Δ(rhaD-rhaB)568 ΔlacZ4787(::rrnB-3) hsdR514 rph-1*	(78)
LB2003	*F*^*−*^*kup1* Δ*kdpABC5* Δ*trkA rpsL metE thi rha gal*	(38)
TO114	W3110 (*F*^*−*^*λ*^*−*^*rpoS(Am) rph-1 Inv(rrnD-rrnE)) nhaA*::Km^r^*nhaB*::Em^r^*chaA*::Cm^r^	(41)
Δ*trkG*	BW25113 Δ*trkG*	This study
Δ*trkH*	BW25113 Δ*trkH*	This study
Δ*trkG*Δ*trkH*	BW25113 Δ*trkG*Δ*trkH*	This study
Δ*ghu* (*kdp*^+^)	BW25113 Δ*trkG*Δ*trkH*Δ*kup*	This study
Δ*dhu* (*trkG*^+^)	BW25113 Δ*trkH*Δ*kdpA*Δ*kup*	This study
Δ*dgu* (*trkH*^+^)	BW25113 Δ*kdpA*Δ*kup*Δ*trkG*	This study
Δ*dgh* (*kup*^+^)	BW25113 Δ*trkG*Δ*trkH*Δ*kdpA*	This study
Δ*dghu* (Δ4)	BW25113 Δ*trkG*Δ*trkH*Δ*kup*Δ*kdpA*	This study
Δ*abc*	BW25113 Δ*nhaA*Δ*nhaB*Δ*chaA*	This study
Δ*abcg*	BW25113 Δ*nhaA*Δ*nhaB*Δ*chaA*Δ*trkG*::Km^r^	This study
Δ*abch*	BW25113 Δ*nhaA*Δ*nhaB*Δ*chaA*Δ*trkH*::Km^r^	This study
W3110	F^*−*^ λ^−^ rph-1 IN(rrnD-rrnE)1	(86)
Δ*hns*	W3110 Δ*hns*	(55)
Δ*hns*Δ*stpA*	W3110 Δ*hns*::Km^r^Δ*stpA*::Cm^r^	(55)
Δ*hns*Δ*ydgT*	W3110 Δ*hns*::Km^r^Δ*ydgT*	(55)
Δ*hns*Δ*hha*	W3110 Δ*hns*::Km^r^Δ*hha*	(55)

Abbreviations: Cm, chloramphenicol; Em, erythromycin; Km, kanamycin.

### Na^+^ activates TrkG-mediated K^+^ uptake

**Figure 2:**
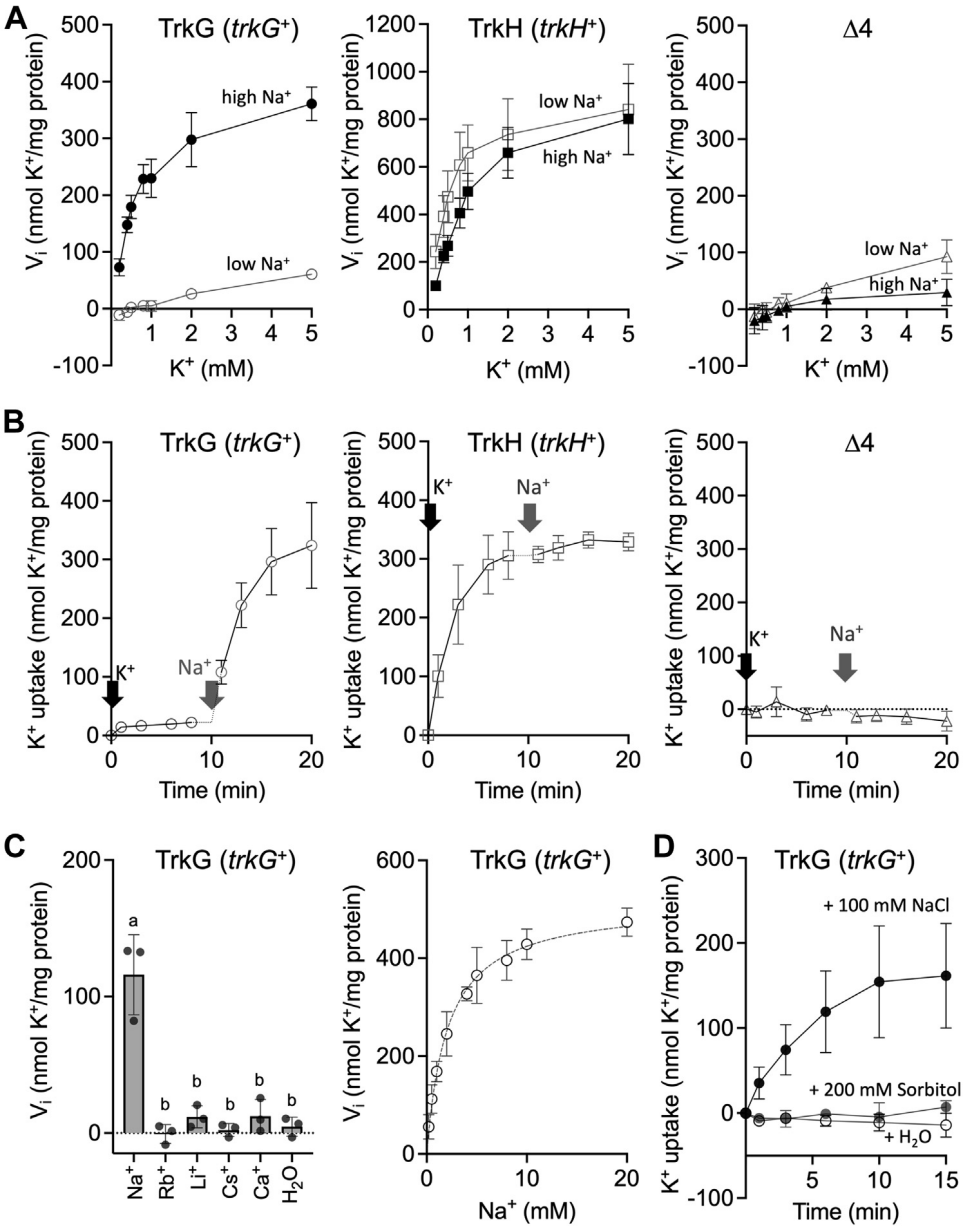
**Na**^**+**^**dependency of TrkG-mediated K**^**+**^**uptake activity.***A*, initial velocity of K^+^ uptake was measured during the first minute after the addition of 0.2 to 5 mM KCl to cells resuspended in 200 mM Hepes–NaOH (pH 7.5) (*closed symbols*) (containing 149.7 mM Na^+^ measured by atomic absorption spectrometry) or Hepes–triethanolamine (pH 7.5) (*open symbols*) (containing 7.7 μM Na^+^ measured by atomic absorption spectrometry). Δ*dhu* (*trkG*^+^) (*circles*), Δ*dgu* (*trkH*^+^) (*squares*), and Δ*dghu* (Δ4) (*triangles*) were used to measure the K^+^ uptake activity of TrkG, TrkH, and negative control, respectively. *B*, K^+^ content determined in cells suspended in 200 mM Hepes–triethanolamine. Addition of 0.1 mM KCl (*t* = 0 min) to the cells was followed by the addition of 1 mM NaCl (*t* = 10 min). *C*, initial velocity of K^+^ uptake was measured during the first minute after the addition of 1 mM KCl to the cell resuspended in 200 mM Hepes–triethanolamine buffer (pH 7.5) supplemented with 1 mM NaCl, RbCl, LiCl, CsCl, or CaCl_2_ (*left*) or with 0.2 to 20 mM NaCl (*right*). One-way ANOVA, Tukey test, *p* < 0.05. Different letters above the bar in graph means statistically different. *D*, the K^+^ content was measured in cells treated by adding 100 mM NaCl (*black symbols*), 200 mM sorbitol (*gray symbols*), or water (*white symbols*) 10 min before the addition of 0.1 mM KCl (*t* = 0 min). Data obtained from three experiments, biological replicates. Mean ± SD. *N* = 3. *Solid line* (*A*, *B*, and *D*) connects means, whereas the *dotted line* (*C*) shows the Michaelis–Menten nonlinear regression curve fit. K^+^, potassium ion; Na^+^, sodium ion.

Some members of the Trk/Ktr/HKT family of transporters have Na^+^-dependent K^+^ uptake activity (30, 39, 40). However, it remains unclear whether K^+^ transport mediated by TrkG and TrkH is also influenced by Na^+^. The Na^+^ dependency of TrkG and TrkH was examined by comparing the K^+^ uptake of Δ*dhu* (*trkG*^+^) and Δ*dgu* (*trkH*^+^) in high-Na^+^ buffer (Hepes–NaOH, 149.7 mM Na^+^ measured by atomic absorption spectrometry) *versus* low-Na^+^ buffer (Hepes–triethanolamine, 7.7 μM Na^+^ measured by atomic absorption spectrometry). K^+^ uptake activity of Δ*dhu* (*trkG*^+^) was significantly higher in high-Na^+^ buffer, compared with that in low-Na^+^ buffer, whereas the K^+^ uptake activity of Δ*dgu* (*trkH*^+^) was similar in both buffers (Fig. 2*A*). To further corroborate Na^+^ dependence of TrkG, we measured the K^+^ uptake rate of Δ*dhu* (*trkG*^+^) in low-Na^+^ buffer while adding K^+^ followed by Na^+^. The Δ*dhu* (*trkG*^+^) strain accumulated K^+^ very slowly in the presence of 0.1 mM K^+^, but the subsequent addition of 1 mM Na^+^ increased intracellular K^+^ accumulation dramatically (Fig. 2*B*, *left*). In contrast, the Δ*dgu* (*trkH*^+^) strain showed high K^+^ uptake once 0.1 mM K^+^ was added, independently of Na^+^ (Fig. 2*B*, *center*). Next, we examined the effect of other cations on the activation of TrkG (Fig. 2*C*, *left*). Rb^+^, Li^+^, Cs^+^, and Ca^2+^ did not activate K^+^ uptake in Δ*dhu* (*trkG*^+^). Therefore, Na^+^ was the only monovalent cation involved in TrkG activation. The half-activation constant for Na^+^ activation of TrkG-mediated K^+^ uptake was calculated to be 2.1 mM (Fig. 2*C*, *right*). Since high concentrations of NaCl also increase the osmotic pressure, we also considered the possibility that osmotic pressure may be involved in TrkG activation. We therefore examined the effect of high osmolarity on TrkG (Fig. 2*D*). Activation after addition of 200 mM sorbitol was smaller than activation by 100 mM NaCl, indicating that Na^+^ itself enhanced TrkG-mediated K^+^ transport.

**Figure 3:**
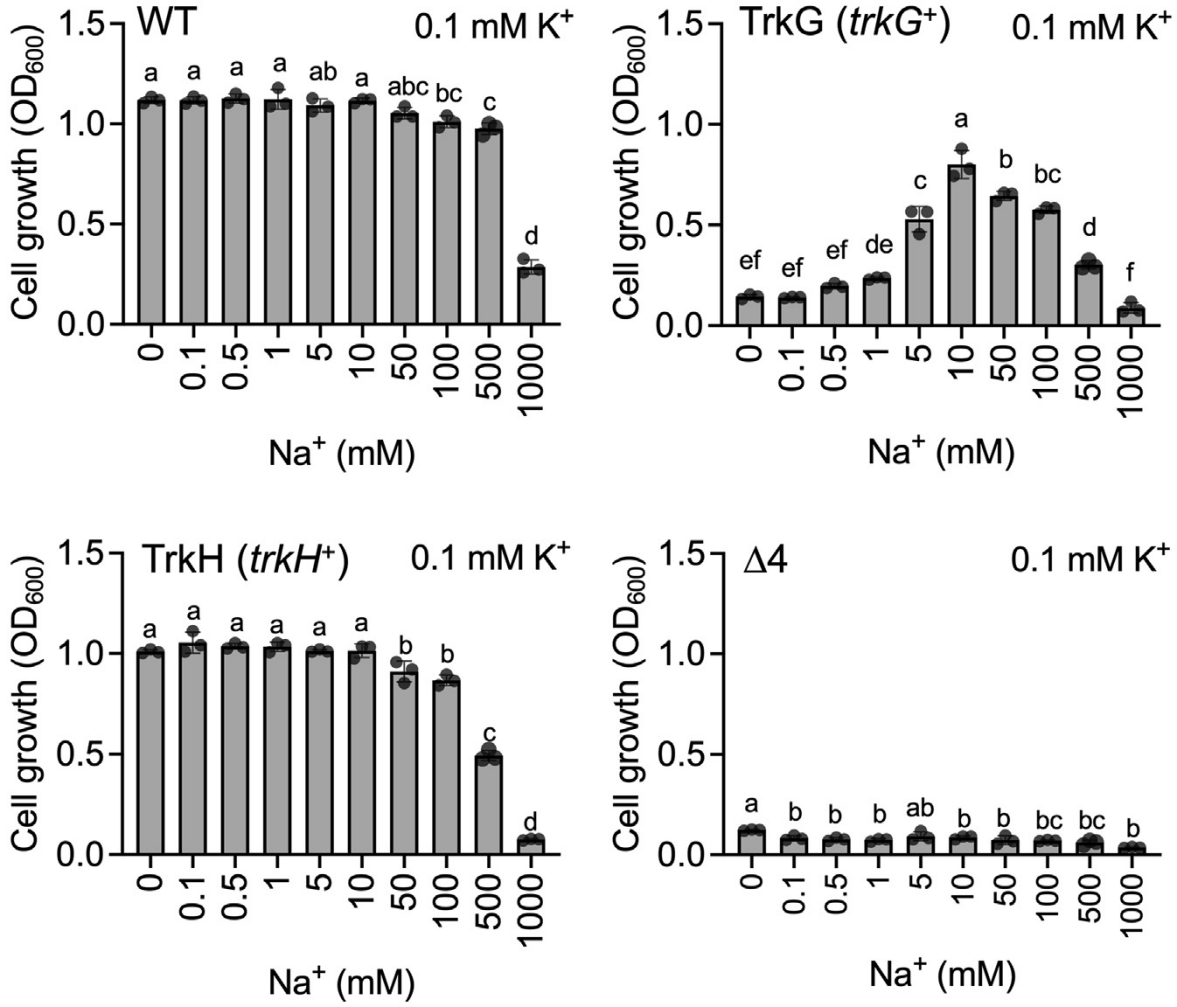
**Stimulation of growth of Δ*dhu* (*trkG***^***+***^**) through addition of Na**^**+**^**.** Growth assay of *Escherichia coli* BW25113 WT, Δ*dhu* (*trkG*^+^), Δ*dgu* (*trkH*^+^), and Δ*dghu* (Δ4) in phosphoric acid–based medium (containing K^+^ 17 μM K^+^ and 33 μM Na^+^ measured by atomic absorption spectrometry) supplemented with 0.1 mM KCl and various concentrations of NaCl. Cell growth was determined after an overnight incubation (*t* = 15 h) at 30 °C. Mean ± SD. n = 3, biological replicates. One-way ANOVA, Tukey test, *p* < 0.05. *Different letters* above the bar in graph means statistically different. K^+^, potassium ion; Na^+^, sodium ion.

To evaluate the effect of Na^+^ on TrkG and TrkH *in vivo*, growth of Δ*dhu* (*trkG*^+^) and Δ*dgu* (*trkH*^+^) was compared in phosphoric acid–based medium containing 0.1 mM KCl and 0 to 1000 mM NaCl (Fig. 3). The basal medium already contained trace concentrations of 17 μM K^+^ and 33 μM Na^+^ as measured by an atomic absorption spectrometry. The Δ*dghu* (Δ4) mutant was unable to grow in any of these media because of the low K^+^ concentration. The growth of Δ*dhu* (*trkG*^+^) increased with increasing Na^+^ in the medium and peaking at 10 mM Na^+^. These data supported the conclusion that TrkG-mediated K^+^ uptake was enhanced by Na^+^. However, concentrations of Na^+^ above 10 mM inhibited growth. It is likely that this impairment of cell growth is due to excess Na^+^ taken up by TrkG.

### TrkG mediates Na^+^ uptake but not Na^+^ efflux

**Figure 4:**
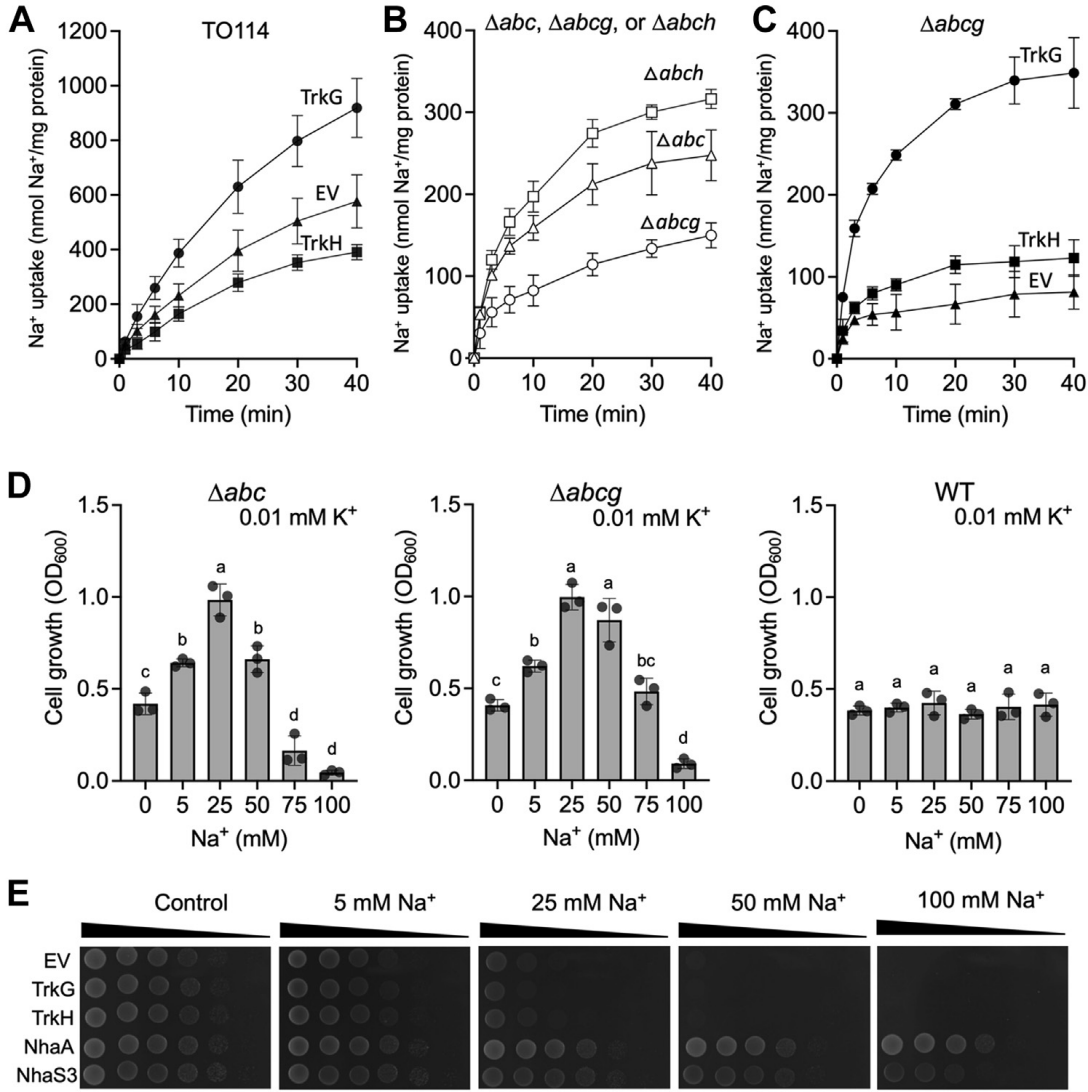
**Mediation of Na**^**+**^**uptake by TrkG.***A*–*C*, to measure Na^+^ content, cells were incubated in 200 mM Hepes–triethanolamine (pH 7.5) supplemented with 10 mM glucose, followed by the addition of 1 mM NaCl (*t* = 0 min). *A*, *Escherichia coli* strain TO114 transformed with TrkG (*closed circles*), TrkH (*closed squares*), and empty vector (*closed triangles*). *B*, *E. coli* mutant strains Δ*abcg* (*open circles*), Δ*abch* (*open squares*), and Δ*abc* (*open triangles*). *C*, Δ*abcg* transformed with same plasmids as in (*A*). *D*, growth assay of *E. coli* Δ*abc*, Δ*abcg*, and WT in phosphoric acid–based medium supplemented with 0.01 mM KCl and various concentrations of NaCl. Cell growth was determined after an overnight incubation (*t* = 15 h) at 30 °C. Mean ± SD. n = 3, biological replicates. One-way ANOVA, Tukey test, *p* < 0.05. *Different letters* above the bars in the graph means statistically different. *E*, growth test of TO114 expressing *E. coli* TrkG, TrkH, NhaA, or *Synechocystis* sp. PCC6803 NhaS3 on KLB medium (control) supplemented with various NaCl concentrations. Ten-fold serial dilutions of the cells were spotted onto agar medium and then incubated at 26 °C for 1 day. The experiment was repeated three times; representative results are shown. Na^+^, sodium ion.

We explored the ability of TrkG to transport Na^+^ by measuring of Na^+^ accumulation inside the cells of *E. coli* TO114 (41), a strain lacking the activity of three Na^+^ efflux–Na^+^/H^+^ antiporters, NhaA, NhaB, and ChaA. The cells expressing TrkG accumulated more intracellular Na^+^ than those expressing TrkH or containing an empty vector (Fig. 4*A*), indicating that TrkG had Na^+^ uptake activity. Since the background activity of Na^+^ uptake in TO114 was high, it is likely that endogenous TrkG contributed to this background activity. To confirm this hypothesis, we constructed quadruple knockout mutants of Δ*nhaA*Δ*nhaB*Δ*chaA*Δ*trkG* (Δ*abcg*) as well as Δ*nhaA*Δ*nhaB*Δ*chaA*Δ*trkH* (Δ*abch*) in the BW25113 background as well as Δ*nhaA*Δ*nhaB*Δ*chaA* (Δ*abc*) as a control (Table 1). Na^+^ uptake activity was significantly lower in Δ*abcg*, compared with the control strain Δ*abc* or Δ*abch* (Fig. 4*B*). Reintroduction of *trkG* into Δ*abcg* successfully restored Na^+^ uptake activity of the mutant (Fig. 4*C*). This result suggested that TrkG conferred most of the Na^+^ uptake activity in Δ*abc* background strains, whereas TrkH did not conduct Na^+^ influx. To re-examine the effect of Na^+^ uptake by TrkG on the growth of *E. coli*, we compared the growth of Δ*abc* and Δ*abcg* at low K^+^ medium with added Na^+^. Growth of the Δ*abcg* strain was less sensitive to higher Na^+^ concentrations (50–100 mM) (Fig. 4*D*). This result is consistent with the interpretation that TrkG-mediated Na^+^ influx led to increased Na^+^ toxicity at high Na^+^. The growth of Δ*abc* increased with increasing Na^+^ concentrations and showed optimum at 25 mM Na^+^, which was not observed in the wildtype. This seems unlikely to be related to TrkG, but the actual reasons remain unclear. We further examined whether TrkG had Na^+^ efflux activity using a plate assay. If TrkG or TrkH mediated Na^+^ efflux, transformed cells should become more Na^+^ tolerant and therefore able to grow in medium with higher Na^+^. The positive controls, *E. coli* NhaA (42) and *Synechocystis* NhaS3 (43), grew well on Na^+^-supplemented medium. The growth of the cells expressing TrkG and TrkH was inhibited as more Na^+^ was added to the medium (Fig. 4*E*). None of our results indicated that TrkG was involved in alleviation of salinity stress.

### D327 might be important for Na^+^ dependency of TrkG

**Figure 5:**
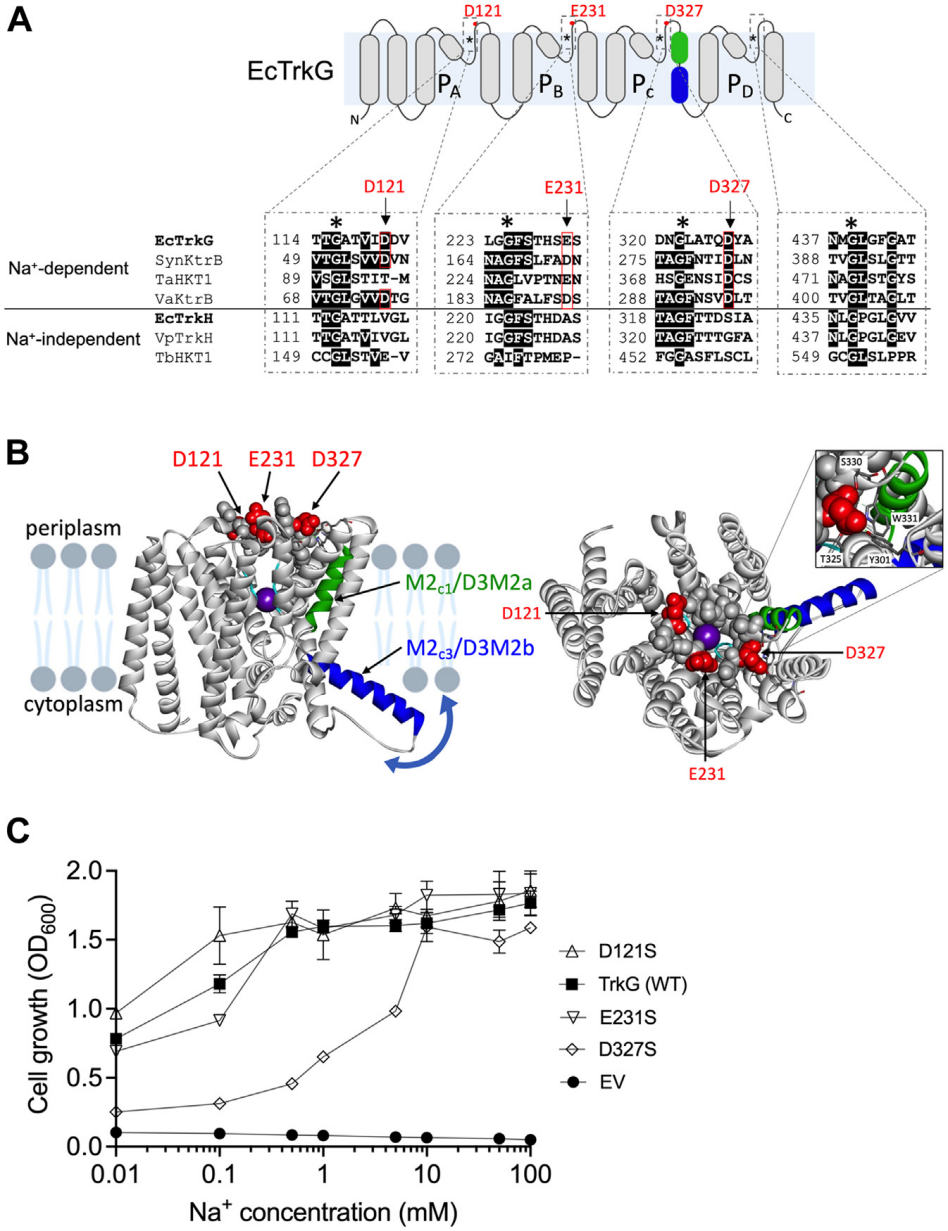
**Negatively charged residues near conserved glycines of TrkG.***A*, schematic illustration showing *E. coli* TrkG structure with the location of the negative residues (*red dots*) near the first, second, and third conserved glycine. *Green* indicates M2_c1_/D3M2a, and *blue* indicates M2_c3_/D3M2b. The conserved glycines that function as K^+^ selectivity filters are indicated by an *asterisk*. The *insets* show amino acid sequence alignments of the P-loop area of *Escherichia coli* TrkG (NP_415881.1), *Synechocystis* sp PCC6803 KtrB (BAL35525.1), *Triticum aestivum* HKT1 (Q41515), *Vibrio alginolyticus* KtrB (O87953), *E. coli* TrkH (YP_026273.1), *Vibrio parahaemolyticus* TrkH (Q87TN7) and *Trypanosoma brucei* HKT1 (Tb927.10.4300). Conserved residues are shaded with *black*. The *line* separates the Na^+^-activated Trk/Ktr/HKT transporters (*top*) from Na^+^-independent Trk/Ktr/HKT transporters (*bottom*). *B*, model structure of TrkG (AF-P23849). The negatively charged residues (D121, E231, and D327) and other residues at the periplasmic gate are represented by *red* and *gray spheres*, respectively, in both the *side* (*left*) and the *top* (*right*) view. The putative motion of M2_c3_/D3M2b is indicated by the *arrow*, and the *inset* illustrates the interactions of D327. The K^+^ ion (*purple sphere*) was associated with the critical glycine. *C*, growth assay of *E. coli* BW25113 Δ*dghu* (Δ4) overexpressing empty vector (EV), WT TrkG, TrkG^D121^, TrkG^E231S^, and TrkG^D327S^ in phosphoric acid–based medium supplemented with 1 mM KCl and different concentrations of NaCl. Cell growth was determined after an overnight incubation (*t* = 15 h) at 30 °C. Mean ± SD, n = 3, biological replicates. Na^+^, sodium ion; K^+^, potassium ion.

Our data (Fig. 2) show that TrkG required Na^+^ for its K^+^ transport activity, whereas TrkH did not. Such different characteristics related to Na^+^ independence are not uncommon in Trk/Ktr/HKT-type transporters (40, 44, 45), but the specific structures responsible for these differences remain unknown. To identify which part of the TrkG protein was responsible for its Na^+^ dependency, we tested several combinations of TrkG and TrkH variants and several chimeras, but all of them showed loss of K^+^ uptake ability (see Discussion section and Fig. S3*B*). While comparing the amino acid sequence of Na^+^-activated Trk/Ktr/HKT transporters (*Synechocystis* KtrB (30), *Triticum aestivum* or wheat HKT1 (40), *V. alginolyticus* KtrB (39)) and Na^+^-independent transporters (*Vibrio parahaemolyticus* TrkH (22) and *Trypanosoma brucei* HKT1 (45)) (Fig. 5*A*), we focused on three negatively charged residues (D121, E231, and D327) that are located five or six residues away from the most important glycine in the first, second, and third K^+^ selectivity filter in the pore regions (29, 46, 47) and are conserved in Na^+^-activated transporters but missing in Na^+^-independent transporters. The model structure of TrkG (obtained from the AlphaFold Protein Structure Database: AF-P23849-F1; https://alphafold.ebi.ac.uk (48)) revealed that these three residues are located near the selectivity filter, facing the periplasmic region (Fig. 5*B*). We replaced the negatively charged residues with the neutral amino acid, serine, to create three TrkG variants, TrkG^D121S^, TrkG^E231S^ and TrkG^D327S^. Some variants had very low K^+^ uptake activity and low amounts of the protein, which made it difficult to assess their Na^+^ dependency. Thereby, as an alternative approach, the viability of Δ*dghu* (Δ4) cells expressing individual variants was examined in phosphoric acid–based medium (Fig. 5*C*). TrkG^D121S^ and TrkG^E231S^ grew as well as the wildtype. In contrast, the growth of cells expressing TrkG^D327S^ was much lower than that of the wildtype at low Na^+^ but was similar to the wildtype at 10 to 100 mM Na^+^. This reflected that replacing D327 with serine increased the Na^+^ requirement for TrkG-containing cells, suggesting that D327 was crucial for Na^+^-dependent activation of TrkG-mediated K^+^ transport.

### Regulation of *trkG* expression

**Figure 6:**
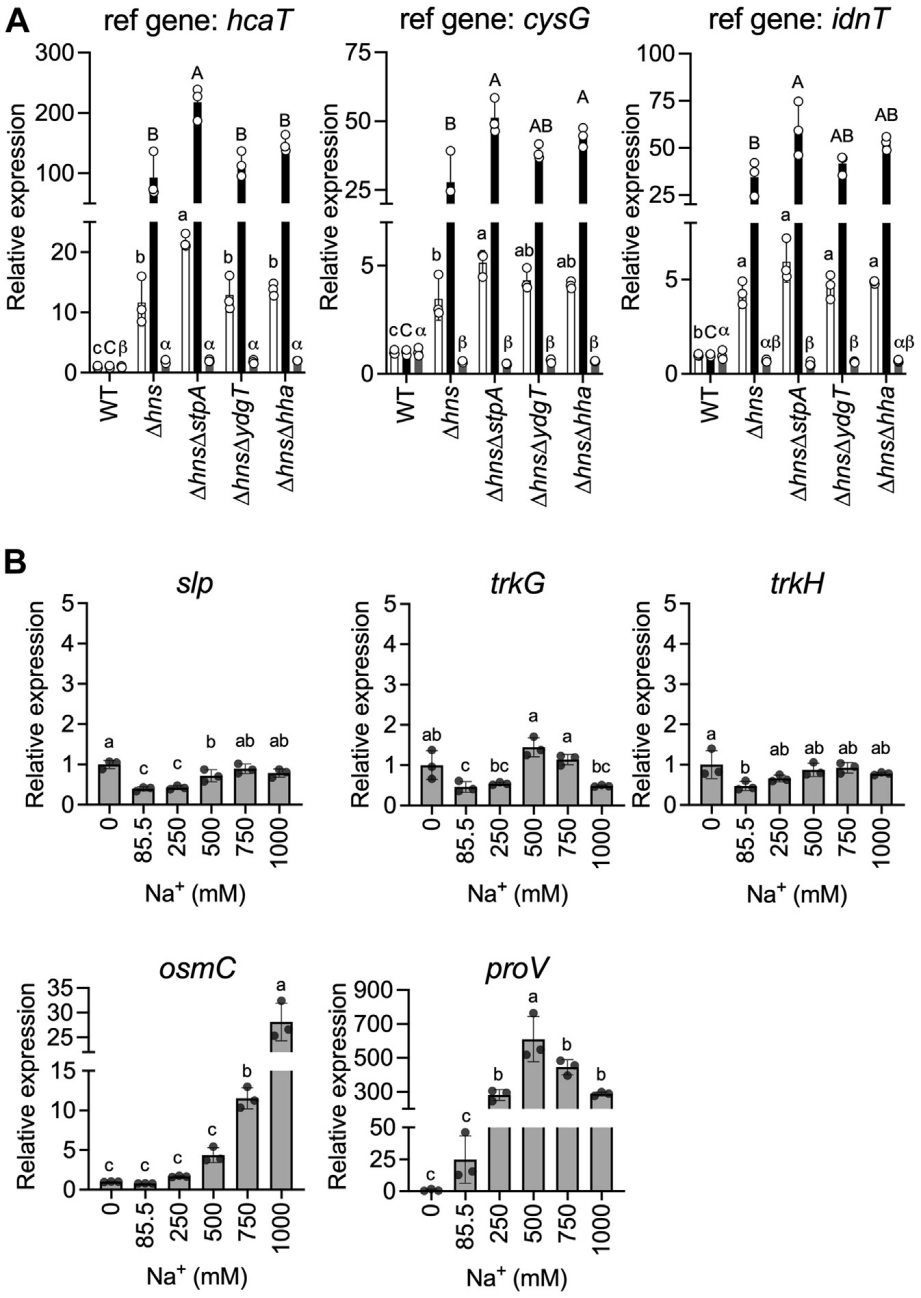
***trkG* expression is repressed by H-NS and not induced by osmotic stress.***A*, relative expression levels of *slp* (*white bars*), *trkG* (*black bars*), and *trkH* (*gray bars*) normalized to the indicated reference genes in *Escherichia coli* W3110 WT, Δ*hns*, Δ*hns*Δ*stpA*, Δ*hns*Δ*ydgT*, and Δ*hns*Δ*hha* grown in the minimal medium supplemented with 30 mM KCl. *B*, relative expression levels of *slp*, *trkG*, *trkH*, *osmC*, and *proV* normalized to *hcaT* in *E. coli* W3110 WT grown in LB containing NaCl at the listed concentrations (85.5 mM NaCl was included because it is the approximate NaCl concentration in widely used LB medium). The relative expression level for each gene was calculated in relation to WT (*A*) or 0 mM NaCl medium (*B*), set at an arbitrary value of 1. Mean ± SD. n = 3, biological replicates. One-way ANOVA, Tukey test, *p* < 0.05. Different letters above the bar in graph means statistically different. H-NS, histone-like nucleoid structuring protein or heat-stable nucleoid-structuring protein.

Because *trkG* has been proposed to be a “foreign” gene in *E. coli* and may be negatively regulated by H-NS (33, 36), we compared the level of *trkG* transcript in Δ*hns* and wildtype by quantitative RT–PCR (qRT–PCR) using three reference genes, *hcaT*, *cysG*, and *idnT* whose expression is stable under various conditions (49). We included *slp* whose expression is under the control of H-NS as an experiment control (50, 51). Expression of *slp* was higher in the Δ*hns* strain compared with the wildtype, regardless of the reference gene used (Fig. 6*A*). The *trkG* expression was significantly higher in Δ*hns*, compared with the wildtype, suggesting that H-NS negatively regulated *trkG* expression (Fig. 6*A*). In contrast, *trkH* expression remained unchanged in all strains, confirming that *trkH* expression was not regulated by H-NS. These results were in agreement with data obtained by ChIP-chip analysis (36). *E. coli* has an H-NS paralog, StpA (52), which plays a role as a molecular backup for H-NS (53). Hha and YdgT proteins interact with either H-NS or StpA to modulate expression in *E. coli* (54, 55). As expected, *trkG* expression was increased in double knockout mutants of *hns* and *stpA* (Δ*hns*Δ*stpA*), indicating that *stpA* may also repress *trkG* expression (Fig. 6*A*). H-NS targeted genes are often regulated by environmental factors including osmotic stress (56, 57). In addition, some Trk/Ktr/HKT family proteins are involved in high osmolarity stress adaptation (30, 58). Therefore, we measured the transcript level of *trkG* in *E. coli* grown with varying concentrations of NaCl, using LB medium devoid of NaCl as a based medium (Fig. 6*B*). We included *osmC* and *proV* in the experiments because both genes are not only modulated by H-NS but also induced by osmotic stress (56, 59). We confirmed that the level of *osmC* and *proV* transcripts increased at high concentrations of NaCl in the medium, indicating that the cells adapted to high osmotic stress (Fig. 6*B*). However, the expression levels of *trkG*, *slp*, and *trkH* transcripts were not significantly affected by the concentration of NaCl (0–1000 mM). These results indicated that *trkG* expression was not induced by high osmotic stress although it was in part regulated by H-NS.

### Role of TrkG in *E. coli*

**Figure 7:**
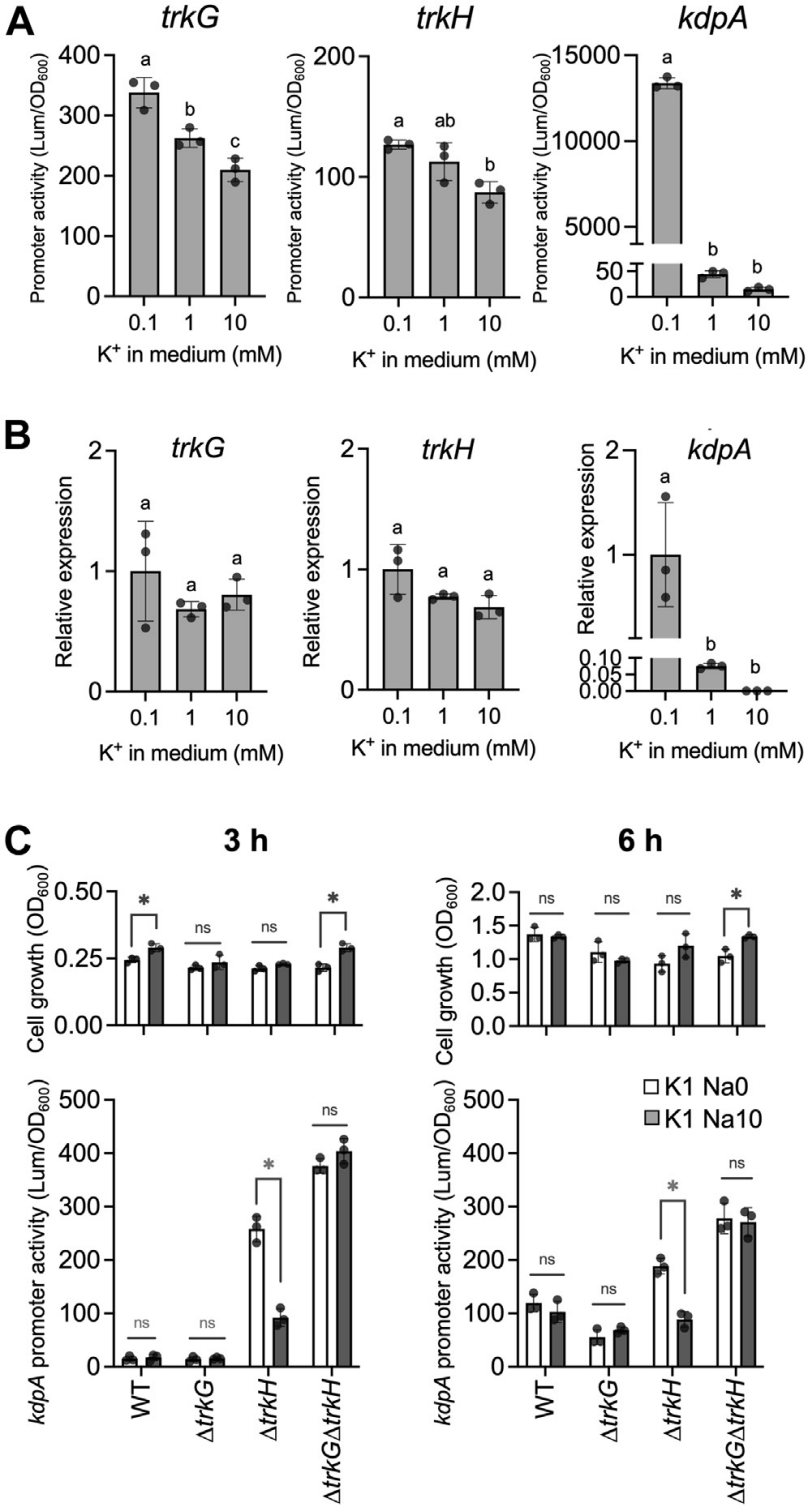
**Contribution of TrkG to K**^**+**^**uptake in the presence of Na**^**+**^**.***A*, promoter activity of *trkG*, *trkH*, and *kdpA*, measured by luciferase reporter assay. Transformants of *Escherichia coli* wildtype were grown in phosphoric acid–based medium supplemented with various concentrations of KCl. Promoter activity is shown as luciferase luminescence normalized to cell growth (absorbance at 600 nm) after an overnight incubation (*t* = 15 h) at 30 °C. *B*, relative expression levels of *trkG*, *trkH*, and *kdpA* normalized to *hcaT* in *E. coli* BW25113 wildtype grown in phosphoric acid–based medium supplemented with various concentrations of KCl. The relative expression level for each gene was calculated in relation to the 0.1 mM KCl medium, set at an arbitrary value of 1. Mean ± SD. n = 3, biological replicates. One-way ANOVA, Tukey test, *p* < 0.05. Different letters above the bar in graph means statistically different. *C*, cell growth (*top*) and normalized promoter activity (*lower*) of *kdpA′*-*lux* in *E. coli* wildtype, Δ*trkG*, Δ*trkH*, and Δ*trkG*Δ*trkH*. Transformants were grown in phosphoric acid–based medium supplemented with 1 mM KCl with (*gray bars*) or without (*white bars*) addition of 10 mM NaCl. Mean ± SD. n = 3, biological replicates. Unpaired *t* test, Holm–Šídák method, *p* < 0.05. *Asterisk* shows *p* < 0.05, ns means not significant. K^+^, potassium ion; Na^+^, sodium ion.

To gain insight into the role of TrkG in *E. coli*, we measured its promoter activity in *E. coli* using a bioluminescent reporter system (Fig. 7*A*) and determined the level of mRNA transcripts in the wildtype by qRT–PCR (Fig. 7*B*). The intensity of luminescence produced by *trkG* promoter–controlled luciferase decreased in high K^+^ medium (Fig. 7*A*, *left*). However, the *trkG* transcript level did not change significantly, regardless of the K^+^ concentration in the medium (Fig. 7*B*, *left*). On the other hand, for TrkH, both promoter activity and transcript level remained relatively stable at various K^+^ concentrations (Fig. 7, *A* and *B*, *center*). The luciferase activity controlled by the *kdpA* promoter was only significantly higher when cells were grown in medium containing 0.1 mM K^+^ or less. At K^+^ concentrations (1 and 10 mM), the *kdpA* promoter activity was very low (approximately 44.44 for 1 mM K^+^ and 14.5 for 10 mM K^+^), indicating there would be some basal expression of *kdpA* at sufficient K^+^ conditions (Fig. 7*A*, *right*). This result was consistent with the data obtained by qRT–PCR (Fig. 7*B*, *right*). To understand what potential benefit TrkG may provide for *E. coli*, we compared the growth of wildtype *E. coli*, Δ*trkG*, and Δ*trkH* under various stress conditions but were unable to identify specific conditions where the growth of Δ*trkG* was either inhibited or improved. To further elucidate the connection of TrkG with TrkH, we constructed mutants expressing the bacterial luciferase gene under control of the *kdpA* promoter. We then determined the *kdp* gene expression level in the mutants as a way to monitor the intracellular K^+^ content. When the cytosolic K^+^ content is low, expression of *kdpA* is induced to replenish K^+^ from the external space (60). *E. coli* wildtype, Δ*trkG*, Δ*trkH*, and Δ*trkG*Δ*trkH* showed similar growth in a phosphoric acid–based medium supplemented with 1 mM K^+^ regardless of the presence/absence of Na^+^ (Fig. 7*C*, *top panels*). The bioluminescence intensity of the luciferase remained at a relatively low level in the wildtype and Δ*trkG* (Fig. 7*C*, *bottom panels*) at 3 h. TrkH probably functioned in both strains under these conditions; therefore, activation of the *kdp* promoter was not needed. In Δ*trkH*, bioluminescence intensity was reduced to 36% at 3 h (47% at 6 h) in medium with added Na^+^, compared with medium without added Na^+^, indicating that in the presence of Na^+^, the Na^+^-stimulated TrkG activity was able to contribute to the replenishment of K^+^ (Fig. 7*C*). In the double mutant, Δ*trkH*Δ*trkG*, bioluminescence increased both in medium with and without Na^+^. These results indicated that TrkH was the major Trk transporter in *E. coli*, and that TrkG could support replenishment of K^+^ when Na^+^ was present in the medium.

## Discussion

**Figure 8:**
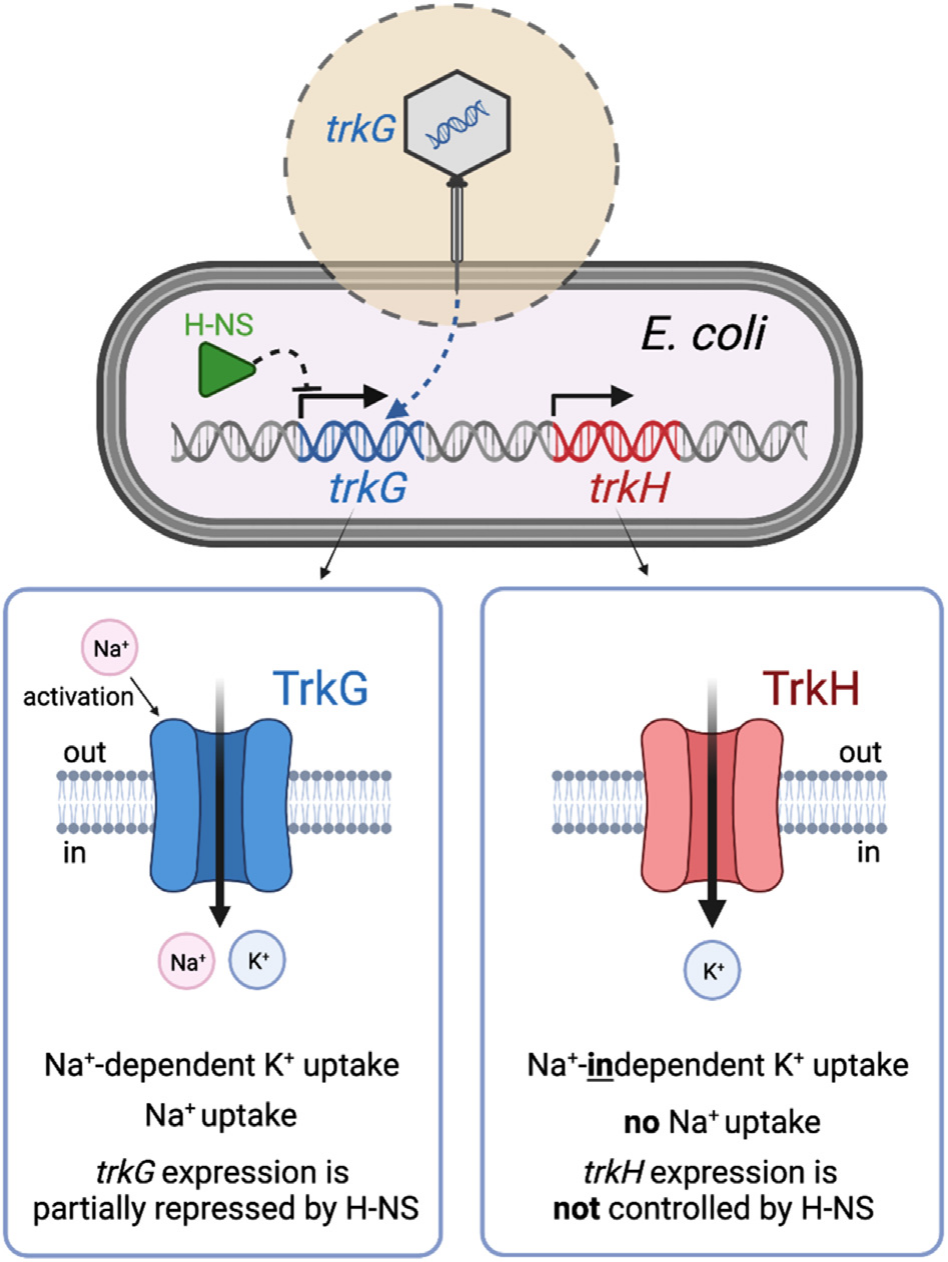
**Differentcharacteristics of *Escherichia coli* K-12 TrkG and TrkH.** The *trkG* gene is presumed to be a “foreign” gene that was acquired through bacteriophage transduction (in the *dashed circle*). H-NS partially represses the expression of *trkG* but not *trkH*. TrkG shows Na^+^-dependent K^+^ uptake and Na^+^ uptake activity while TrkH shows Na^+^-independent K^+^ uptake and no Na^+^ uptake activity. H-NS, histone-like nucleoid structuring protein or heat-stable nucleoid-structuring protein; K^+^, potassium ion; Na^+^, sodium ion.

This study comprehensively investigated the function, physiological role, and genetic background of Trk transporters in *E. coli*, yielding these new results: (1) differences in Na^+^ requirement and Na^+^ permeability of TrkG and TrkH, (2) TrkH as the major K^+^ uptake system and TrkG and Kup as minor K^+^ uptake systems before having to use the energy-consuming Kdp-mediated K^+^ absorption, (3) identification of an aspartic acid residue (D327) involved in Na^+^-dependent activation of TrkG, (4) differences in gene regulation of *trkG* and *trkH* by H-NS (summarized in Fig. 8). Our results contribute to a fundamental understanding of Trk/Ktr/HKT-type transporters from prokaryotes to eukaryotes, which are related by common ancestry. Animal and plant cells contain a number of functional K^+^ channels (61, 62), whereas in *E. coli*, the central role of K^+^ uptake is performed by TrkG, TrkH, Kup, and Kdp instead of K^+^ channels. Previously, little attention had been paid to functional differences between TrkG and TrkH. In fact, TrkG and TrkH share basically the same transmembrane topology and the same cytosolic TrkA for their transport activity (18, 20, 23). In *B. subtilis*, which like *E. coli* K-12 also has two Trk/Ktr/HKT-type K^+^ transporters, KtrAB and KtrCD, these are involved in adaptation to osmotic stress (58). However, no differences in the function and physiological roles of KtrAB and KtrCD have been reported. This study revealed that Na^+^-dependent activation of TrkG was one notable feature that was different from TrkH (Fig. 2). The half-activation constant for Na^+^ activation of TrkG was approximately 2.1 mM Na^+^ (Fig. 2*C*). Na^+^-dependent K^+^ uptake of Trk/Ktr/HKT family transporters has been reported for *Synechocystis* KtrABE (30), *V. alginolyticus* KtrAB (39), and wheat HKT1 (40).

To narrow down which part of TrkG was responsible for its Na^+^-dependent activity, we introduced targeted mutations into the pore region and created various chimera variants (Fig. S3). TrkG and TrkH each have four sets of the pore loop between two transmembrane domains (D1–D4). Each pore loop contains a conserved glycine, which is the equivalent of the first glycine of the GYG signature sequence of K^+^ channels (29, 46, 47). We predicted that the amino acid sequence adjacent to the glycine might be involved in Na^+^ dependency (Figs. S2 and S3*A*). We therefore swapped these four residues including the glycine between TrkG and TrkH and tested the K^+^ transport activity of these variants. However, none of them showed any K^+^ uptake activity. We also constructed several chimeras by swapping domains between TrkG and TrkH (Fig. S3*B*), but none of the cultures expressing these chimeras were able to grow in low K^+^ medium (Fig. S3*C*). Thereby, as an alternative approach, we compared the amino acid sequences of Trk/Ktr/HKT-type K^+^ transporters with different properties and noticed negatively charged residues conserved in the transporters, which were absent from Trk/Ktr/HKT with Na^+^-independent activity (Fig. 5*A*). Conversion of one of those residues, D327, located in the third pore region of TrkG, to serine, an amino acid with neutral charge, resulted in a transporter that required higher Na^+^ concentrations than the wildtype (Fig. 5*B*). D327 is located close to a transmembrane domain (referred to as M2_C_/D3M2) (22, 27), which is responsible for gating control (63, 64). From a structural point of view, D327 is interacting with the residues Y301, T325, S330, and W331 that are all located in the vicinity of the N-terminal region of M2_C_/D3M2 (Fig. 5*B*, *inset*). These interactions might affect Na^+^-dependent gating through the motion of the M2_C3_/D3M2b helix (21, 63, 64). Association of Na^+^ with D327 may regulate K^+^ conductance through TrkG. Na^+^ activation is also found in K^+^ channels, for example, Slo2.2, a neuronal Na^+^-activated K^+^ channel (65). It has been reported that a negatively charged residue, aspartic acid, is important for Na^+^-dependent activation of G protein–gated inwardly rectifying K^+^ channels (66, 67). Na^+^ may function as a regulator of some classes of K^+^ transport systems in general (5).

Another distinct property of TrkG was its Na^+^ transport activity (Fig. 4, *A*–*C*). Some members of the Trk/Kup/HKT family are known to be Na^+^ permeable, and these may be more directly ancestral to TrkG than to TrkH (26, 31, 32). Several earlier reports have discussed physiological advantages conferred by the Na^+^ transport activity of Trk/Ktr/HKT transporters. Nutritional Na^+^ uptake by the Trk homolog from rice, OsHKT2;1 occurs in roots under K^+^ starvation conditions (68). Other plant Trk/Ktr/HKT transporters (*e.g.*, TaHKT1 and AtHKT1) also provide Na^+^ uptake routes (26, 31, 32, 69). Under K^+^-limited conditions, Na^+^ can function as a K^+^ substitute, and Na^+^ accumulates in a special compartment (*e.g.*, vacuole) to promote cell expansion (70). Na^+^ supports optimal growth in cyanobacteria (71, 72). Here, we only observed negative effects of Na^+^ toxicity because of TrkG-mediated Na^+^ uptake (Fig. 4*D*). However, this was reminiscent of the way that *E. coli* growth can be supported through uptake of the nonessential and cytotoxic cations Rb^+^ and Cs^+^ under K^+^-limited conditions, thereby allowing *E. coli* to expand its habitat (15).

Genetic exchanges between bacterial species or *via* lateral gene transfer from more evolutionarily distant organisms creates functional diversity and provides plasticity in the adaptation to various environments on an evolutionary timescale (73). In *E. coli* K-12, identified foreign genes account for approximately 13% of the genome. The *trkH* gene is native to *E. coli*; however, *trkG* is located within a phage-derived sequence in the *E. coli* genome (33). Because such foreign genes can potentially disrupt physiological functions of the host cell when they are first introduced, the host can suppress the expression of such foreign genes with the help of a transcription factor, H-NS (74). Consequently, expression of *trkG* was negatively regulated by H-NS, whereas *trkH* was not affected (Fig. 6*A*). However, *trkG* was not completely silenced in *E. coli*, and its expression level was similar to that of *trkH* (Fig. 6, *A* and *B*). TrkG and TrkH also share the same auxiliary cytosolic protein, TrkA, which further reflects the successful integration of *trkG* in *E. coli* (14). A foreign gene that is successfully adopted into the genome and the functioning of the cell often contributes to cellular stress tolerance. H-NS has been proposed to be an osmotic stress sensor, and salt concentrations affect the DNA-binding properties of H-NS (75). Excessive salt induces dissociation of the H-NS and DNA bridge complex, thus allowing RNA polymerase to proceed with the transcription process (75). Therefore, we evaluated whether osmotic stress influenced *trkG* expression. Our data showed that *trkG* expression remained relatively constant regardless of the NaCl concentrations, different from *osmC* and *proV*, whose expression is under the control of H-NS (Fig. 6*B*). Our data in Figure 4*D* suggest that the Na^+^ uptake activity of TrkG does not improve Na^+^ salinity tolerance. Therefore, it is likely that *E. coli* partially represses *trkG* expression through H-NS to avoid the excess “unwanted” Na^+^ uptake mediated by TrkG. Because it was difficult to assess directly how much TrkH or TrkG individually contributed to K^+^ supply of the cells, we instead estimated the expression level of *kdp*, whose expression is controlled by intracellular K^+^ concentrations (37) (Fig. 7). The expression level of *kdp* in both single mutants was lower than that of *kdp* in the Δ*trkG*Δ*trkH* double mutant. Therefore, TrkG and TrkH compensated for each other's loss of activity, which could be the reason why *E. coli* has maintained two homologs of Trk-type transporters over the course of evolution. Our analysis also suggested that TrkH exhibited a strong K^+^ uptake system, compared with TrkG and Kup, and likely acted as a major K^+^ uptake system when Kdp was absent (Fig. 1).

This study added to our understanding of the four kinds of K^+^ uptake system in *E. coli*; Kdp is highly specific to K^+^ (76), whereas Kup also allows Cs^+^ transport across the membrane (14, 15). Here, we showed that TrkH is the major K^+^ uptake system, whereas TrkG transports Na^+^ in addition to K^+^. *E. coli* has therefore acquired powerful uptake systems for different alkaline cations (Na^+^, K^+^, Rb^+^, and Cs^+^). The diversity of these four types of transporters enhances the resilience and adaptability of *E. coli* to various environmental conditions.

## Experimental procedures

### Construction of knockout mutants

The *E. coli* strains used in this study are listed in Table 1. For K^+^ uptake experiments, multiple knockout mutants of the genes encoding the transmembrane protein (*kdpA* [symbolized as *d*], *trkG* [*g*], *trkH* [*h*], and *kup* [*u*]) of the *E. coli* K-12 K^+^ uptake systems were constructed using strains from the Keio collection (77). The kanamycin-resistant cassette was removed by using the FLP helper plasmid (pCP20). The marker-less mutant was then used as a parental strain for the next gene deletion. In-frame gene deletions were generated by PCR-based mutagenesis (78). Briefly, an FRT-flanked chloramphenicol resistance cassette was amplified using a set of primers carrying a sequence with homology to a region of the target gene. The parental strain expressing the λ Red recombinase from pKD46 was transformed with the PCR product. Transformants were selected on chloramphenicol-containing medium (25 μg/ml). The chloramphenicol resistance cassette was then removed to create a marker-less mutant. The process was repeated until all desired multiple mutations were accomplished. To analyze the Na^+^ uptake ability of TrkG and TrkH, a Na^+^ efflux transporter mutant (Δ*nhaA*Δ*nhaB*Δ*chaA* or Δ*abc*) was constructed, and *trkG* or *trkH* mutations were introduced into this background (Δ*abcg* and Δ*abch*, respectively). The mutants were constructed using either the same method described previously or using a version of P1 transduction genome modification (79). In the latter approach, the kanamycin cassette region from the appropriate Keio collection strain was transferred into recipient strains to create multiple mutations with the help of the P1 phage. All gene deletions were confirmed by PCR or whole-genome sequencing.

### Plasmid construction

Plasmids and primers used in this study are listed in Table S1 and S2. To measure K^+^ and Na^+^ uptake, *E. coli trkG* and *trkH* were amplified by PCR using a BamHI site–containing forward primer and a PstI site–containing reverse primer. The resulting 1.4 Kb PCR fragment was purified by gel extraction. The purified PCR fragment was cloned into BamHI- and PstI-digested pPAB404 using an In-Fusion HD cloning kit (Takara Bio USA, Inc), yielding plasmids pPAB404-TrkG and pPAB404-TrkH. Construction of selective pore variants and chimera variants was performed by combining PCR fragments using the same In-Fusion technique. Gene expression in pPAB404 was controlled by the *tac* promoter, and protein expression was induced by the addition of 0.1 mM isopropyl-β-d-1-thiogalactoside (76). For the luciferase reporter promoter assay, the promoter DNA fragment was amplified by PCR with genomic DNA of BW25113 as a template using the following primer pairs: trkH_LUX_F and trkH_LUX_R for pLux-trkH; trkG_LUX_F and trkG_LUX_R for pLux-trkG; and kdpA_LUX_F and kdpA_LUX_R for pLux-kdpA. The PCR fragments were cloned into the pLUX vector as previously described (80). The resulting plasmids were verified by DNA sequencing.

### Growth conditions and mediums

*E. coli* BW25113 and its derivative mutants were routinely grown in KLB medium (0.5% yeast extract, 0.5% KCl, and 1% hypopolypeptone). Growth test at different K^+^ concentrations was essentially conducted as previously described with some modifications (15, 81). For growth tests on plates, *E. coli* K^+^ transporter mutants were first pregrown in K^+^ minimal medium (46 mM Na_2_HPO_4_, 23 mM NaH_2_PO_4_, 8 mM (NH_4_)_2_SO_4_, 0.4 mM MgSO_4_, 6 mM FeSO_4_, 10 μg/ml thiamine, and 1% glucose) supplemented with 30 mM KCl. These precultures were incubated at 30 °C, 150 rpm overnight (15 h). Cells were harvested by centrifugation, washed with K^+^-free buffer (46 mM Na_2_HPO_4_, 23 mM NaH_2_PO_4_, and 8 mM (NH_4_)_2_SO_4_) and then resuspended with the same buffer. The absorbance at 600 nm of the cell suspension was measured and adjusted to 0.5. The cell suspension was serially diluted with K^+^-free buffer, and 5 μl aliquots were spotted onto plates (minimal medium plus 1.5% agar) containing varying concentrations of KCl. The plates were incubated at 30 °C for 48 h. When comparing the cell growth of Δ*dghu* (Δ4) transformants, the minimal medium was supplemented with 50 μg/ml ampicillin and 0.1 mM isopropyl β-d-1-thiogalactopyranoside. For the liquid growth test, the resuspended preculture cells were inoculated into minimal medium at an initial absorbance of 0.05 at 600 nm. Cell growth was measured as absorbance at 600 nm after incubation at 30 °C for 15 h. For growth tests at different K^+^ and Na^+^ concentrations, a phosphoric acid–based medium was created, a modification of the defined medium of Collins and Thune (82). This phosphoric acid–based medium contained 8 mM H_3_PO_4_, 8 mM (NH_4_)_2_SO_4_, 0.4 mM MgSO_4_, 6 μM FeSO_4_, 29 μM MnSO_4_.5H_2_O, 12 μM CaCl_2_.2H_2_O, 17 μM ZnSO_4_.7H_2_O, 0.4 μM CuSO_4_.5H_2_O, 0.4 μM CoCl_2_.6H_2_O, 0.73 μM *p*-aminobenzoic acid, 4.9 μM pyridoxine HCl, 1.5 μM thiamine, 1% (w/v) glucose, and 0.2 to 3.0 mM amino acid mixture (2.2 mM l-alanine, 1.4 mM l-arginine, 3 mM l-asparagine, 0.75 mM l-aspartic acid, 0.41 mM l-cysteine, 2 mM l-glutamic acid, 1.3 mM glycine, 0.39 mM l-histidine, 1.9 mM l-isoleucine, 1.9 mM l-leucine, 1.4 mM l-lysine, 0.67 mM dl-methionine, 0.61 mM l-phenylalanine, 0.87 l-proline, 0.48 mM l-serine, 1.7 mM l-threonine, 0.2 mM l-tryptophan, 0.55 mM l-tyrosine, and 2.1 mM l-valine), the pH was adjusted to 7.4 with Tris.

### Measurement of K^+^ uptake

Cation uptake was determined as described previously with some modifications (30, 39). Briefly, *E. coli* mutants were cultured in a minimal medium containing 20 mM KCl at 30 °C, 150 rpm until absorbance at 578 nm reached ±0.6 to 0.9. The culture was harvested by centrifugation and treated with 1 mM EDTA in 120 mM Tris–HCl (pH 8.0) for 30 min at 37 °C. The cells were washed two times using 200 mM Hepes–NaOH or Hepes–triethanolamine (pH 7.5), then resuspended with the same buffer. Note that 200 mM Hepes–NaOH buffer contains 22.6 μM K^+^ and 149.7 mM Na^+^ and 200 mM Hepes–triethanolamine contains 11 μM K^+^ and 7.7 μM Na^+^ as measured by atomic absorption spectrometer. After 20 min of incubation at room temperature, the cell suspension was transferred into a new flask at an absorbance of 3 at 578 nm. About 10 mM of glucose was added into the suspensions prior to K^+^ addition, and K^+^ was added at the indicated final concentration. At designated time points, 1 ml samples were taken and centrifuged through silicone oil (Sigma–Aldrich) to separate the buffer and cells. The buffer was removed, and the cells were disrupted with 5% trichloroacetic acid and heated at 100 °C for 5 min. The K^+^ content extracted from the cells was determined using an atomic absorption spectrometer (iCE 3500 Thermo Fisher Scientific AA Spectrometer), and the total protein content in the cell pellets was measured using a BCA protein assay (Thermo Scientific). The intracellular concentration of K^+^ was calculated as nanomoles of K^+^/mg cellular protein. K^+^ uptake was calculated by subtracting the intracellular K^+^ concentration before K^+^ addition from the concentration at the indicated time after K^+^ addition.

### Measurement of Na^+^ uptake

Na^+^ uptake was determined using the Na^+^ transporter knockout mutant strain (TO114 or Δ*abc* transformants) and its derivatives. The procedure was similar to the one used for the K^+^ uptake experiments except the growth medium used was KLB supplemented with 30 μg/ml ampicillin and 0.1 mM isopropyl-β-d-1-thiogalactoside to induce protein expression, and the buffer used was 200 mM Hepes–triethanolamine (pH 7.5).

### qRT–PCR

RNA was extracted from early exponential cultures of *E. coli* W3110 wildtype and Δ*hns* (unless otherwise indicated) (absorbance at 600 nm = 0.4–0.6) using TRI reagent (Molecular Research Centre, lnc), followed by genomic DNA removal and complementary DNA synthesis using Revertra ace qPCR RT Master Mix with gDNA Remover (TOYOBO CO, LTD). The KAPA SYBR FAST qPCR Master Mix (2×) Kit (KAPA Biosystems) was used for qRT–PCR. The StepOnePlus Real-Time PCR system (Applied Biosystems) was used as the detection system. The composition of the reaction was 0.5 ng complementary DNA, 1× KAPA SYBR FAST qPCR master mix, 200 nM forward primer, and 200 nM reverse primer (Table S2). The reaction conditions were 95 °C for 3 min (95 °C for 3 s and 60 °C for 20 s) repeated 50 times. The temperature was then increased by 1 °C from 60 °C to 95 °C, and the melting temperature (*T*_m_ value) of the amplified fragment DNA was determined. *hcaT*, *cysG*, and *idnT* were used as reference genes (49).

### Luciferase assay

*E. coli* BW25113 wildtype, Δ*trkG*, Δ*trkH*, and Δ*trkG*Δ*trkH* were transformed with pLUX containing the bacterial luciferase gene under control of the *kdp* promoter. Transformants were grown in phosphoric acid–based medium containing 10 mM KCl and 50 μg/ml kanamycin. These precultures were incubated at 30 °C, 150 rpm overnight (15 h). Cells were harvested by centrifugation, washed with K^+^-free phosphoric acid–based buffer (8 mM H_3_PO_4_, 8 mM (NH_4_)_2_SO_4_, pH adjusted to 7.4 by Tris) and then resuspended with the same buffer. The resuspended preculture cells were inoculated into new medium at an initial absorbance of 0.05 at 600 nm. Cell growth (absorbance at 600 nm) and luciferase emission intensity were measured after 3 h and 6 h incubation at 30 °C. The measurements were performed using a multiplate reader (Synergy H1 Microplate Reader; BioTek). Promoter activity was calculated as luciferase emission intensityabsorbance at 600 nm.

## Data availability

All data are contained within the article and the supporting information.

## Supporting information

This article contains supporting information (22, 76, 78, 83, 84, 85).

## Conflict of interest

The authors declare that they have no conflicts of interest with the contents of this article.
