# Supplementary material for: Two Trk/Ktr/HKT-type potassium transporters, TrkG and TrkH, perform distinct functions in Escherichia coli K-12
Source: J Biol Chem. 2022 Dec 29;299(2):102846. doi: 10.1016/j.jbc.2022.102846 (PMC9898762; doi:10.1016/j.jbc.2022.102846)
Supplement: Supplemental Figures S1–S3 and Tables S1, S2 [file mmc1.pdf]

Supporting Information

Two Trk/Ktr/HKT-type potassium transporters, TrkG and TrkH perform distinct functions in *Escherichia coli* K-12

Ellen Tanudjaja[1], Naomi Hoshi[1], Kaneyoshi Yamamoto[2], Kunio Ihara[3],

Tadaomi Furuta[4], Masaru Tsujii[1], Yasuhiro Ishimaru[1] & Nobuyuki Uozumi[1,*]

Material included:

Supplemental Figure S1-S3

Supplemental Table S1-S2

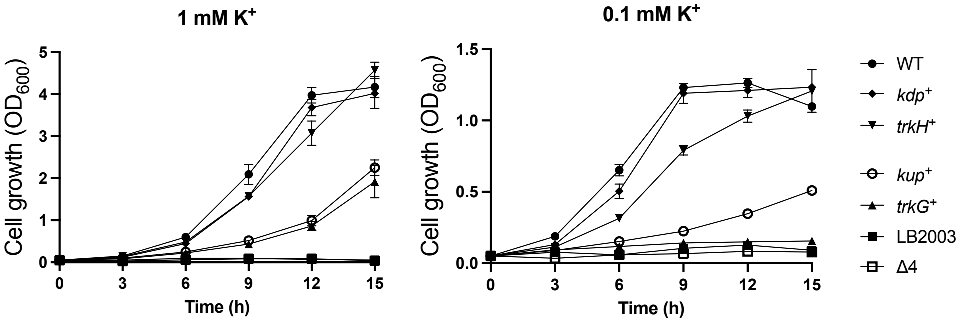

Supplemental figure S1. Growth curves of *E. coli* mutants in K^+ limited medium

Growth of *E. coli* BW25113 wild type and triple knockout mutants at 30 °C. Mean ± SD. *n* = 3, biological replicates. Wild type, WT; LB2003; Δ*ghu* (*kdp*$^+$), Δ*dhu* (*trkG*$^+$), Δ*dgu* (*trkH*$^+$), Δ*dgh* (*kup*$^+$) and Δ*dghu* (Δ4)

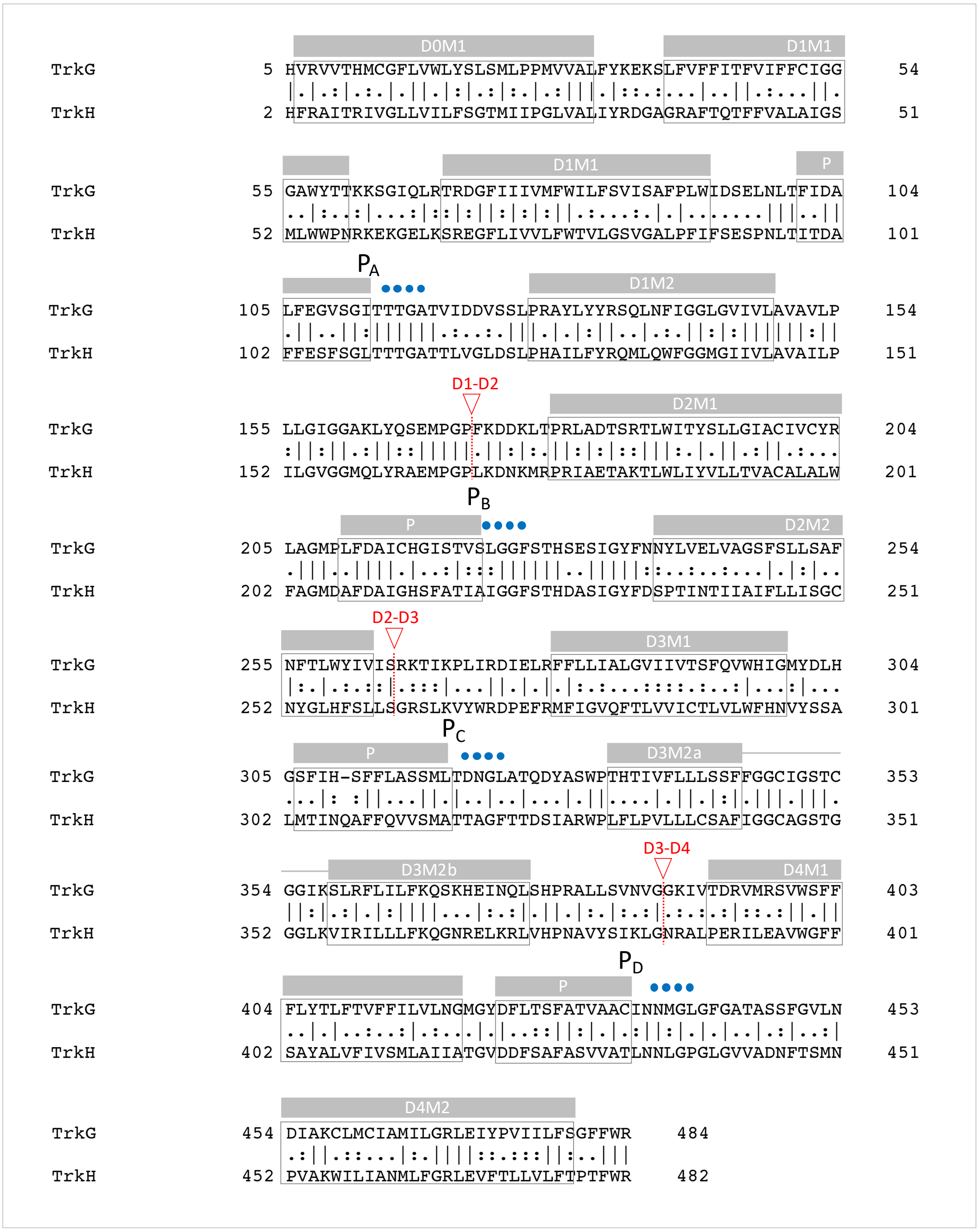

Supplemental Figure S2. Amino acid sequence alignment of TrkG and TrkH.
The amino acid sequences of *E. coli* TrkG (acc# NP_415881) and TrkH (acc#YP_026273) were aligned using EMBOSS Water Pairwise Sequence Alignment https://www.ebi.ac.uk/Tools/psa/emboss_water/ (82). A vertical line (|) indicates a conserved residue, while a colon (:) and a period (.) indicate conservation between groups with strongly or weakly similar properties, respectively. Transmembrane domains were determined based on similarity with the previously analyzed VpTrkH(22). Transmembrane domains are marked with grey bars, P indicates the position of the pore helix. The gray line connecting the two parts of D3M2 (D3M2a and D3M2b) denotes the intramembrane loop. Solid blue circles denote residues forming the K^+ selectivity filter. The open triangle indicates the position of junctions between sections of the chimera protein constructs.

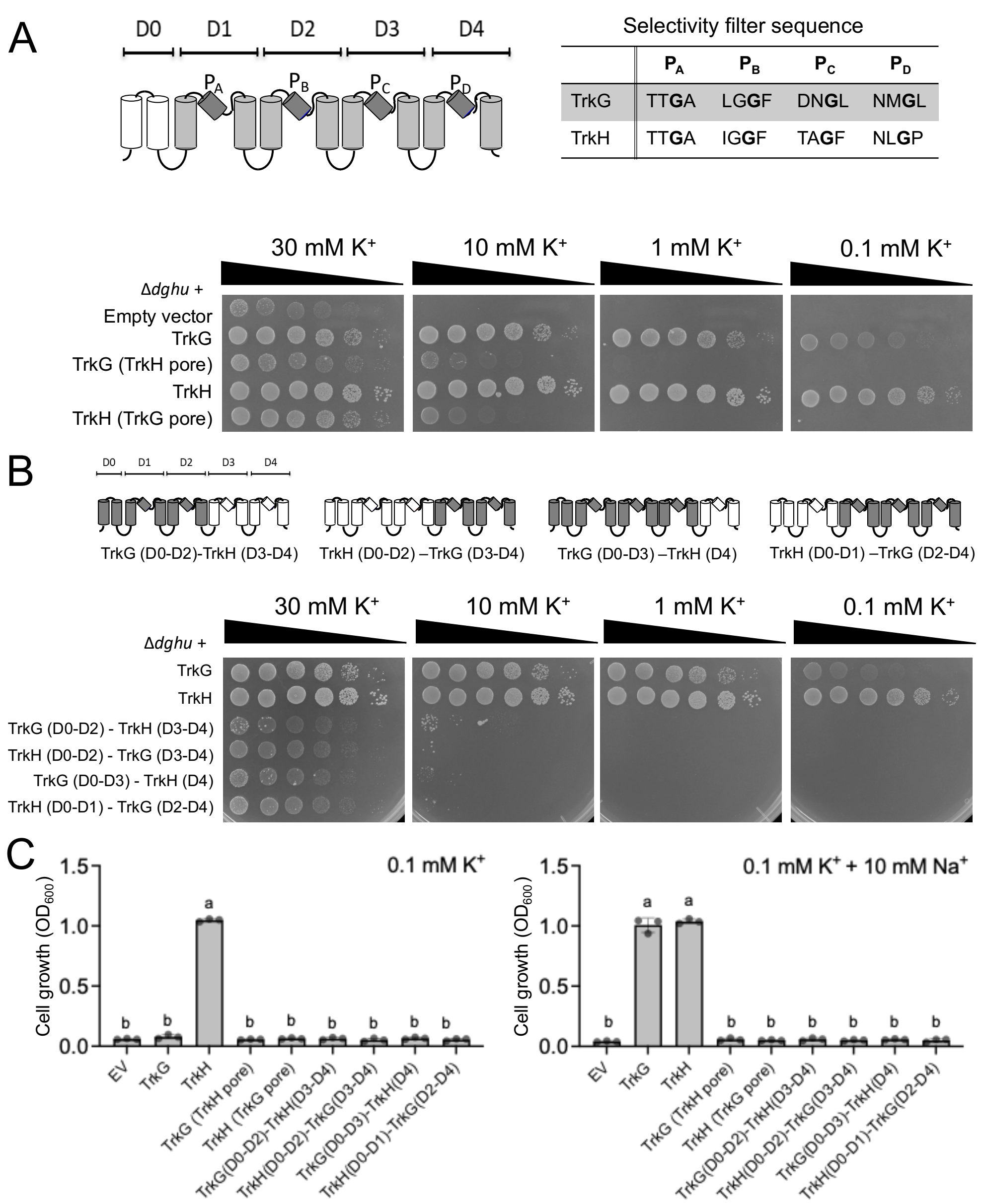

Supplemental Figure S3. Selective pore-variants and chimeras between TrkG and TrkH)
(A) Graphical representation of the TrkG and TrkH protein structure (top left). Sequence comparison of amino acids forming the selectivity filter signature sequence of TrkG and TrkH (22), grouped by their domain locations. The conserved glycine residue is bolded (top right). Growth of *E. coli Δdghu* (Δ4) transformed with plasmids containing TrkG, TrkH or their selective pore-variants on minimal medium supplemented with 30, 10, 1, or 0.1 mM KCl, incubated for 2 d at 30 °C (bottom). (**B**) Graphical representation of chimeras between TrkG and TrkH (top). Growth of *E. coli Δdghu* (Δ4) transformed with plasmids containing TrkG, TrkH or their chimeras on minimal medium supplemented with 30, 10, 1, or 0.1 mM KCl, incubated for 2 d at 30 °C (bottom). (**C**) Growth of *E. coli Δdghu* (Δ4) transformed with plasmids containing TrkG, TrkH or the chimeras. Cells were grown in phosphoric acid-based medium containing 0.1 mM KCl (left) alone or with added 10 mM NaCl (right). Cell growth (OD_{600}) was measured after 15 h incubation at 30 °C. Mean ± SD, $n = 3$, biological replicates. One-way ANOVA, Turkey test, $p < 0.05$. Different letters above the bar in graph means statistically different.

Supplemental Table S1. Plasmids used in this study

Plasmid name	Relevant genotype or description	Reference
pPAB404	expression vector containing P_{tac} promoter and Amp^r	(75)
pPAB404-TrkG	wild-type *trkG*	This study
pPAB404-TrkH	wild-type *trkH*	This study
**Selective pore variants[a]:**		
pPAB404-TrkG (TrkH pore)	*trkG* (L223I-D320T-N321A-L323F-M438L-L440P)	This study
pPAB404-TrkH (TrkG pore)	*trkH* (I220L-T318D-A319N-F321L-L436M-P438L)	This study
**Chimera variants[b]:**		
pPAB404-$TrkG_{(D0-D2)}$-$TrkH_{(D3-D4)}$	*trkG*-S264-*trkH*-G262	This study
pPAB404-$TrkH_{(D0-D2)}$-$TrkG_{(D3-D4)}$	*trkH*-S261-*trkG*-R265	This study
pPAB404-$TrkG_{(D0-3)}$-$TrkH_{(D4)}$	*trkG*-G387-*trkH*-N386	This study
pPAB404-$TrkH_{(D0-D1)}$-$TrkG_{(D2-D4)}$	*trkH*-P168-*trkG*-F172	This study
**Luciferase promoter assay:**		
pLUX	Promoter-less *luxCDABE*	(83)
pLUX-kdpA	pLUX, *kdp'-lux*	This study
pLUX-trkG	pLUX, *trkG'-lux*	This study
pLUX-trkH	pLUX, *trkH'-lux*	This study
**Helper plasmids:**		
pKD3	Template for FRT-flanked Cm cassette	(77)
pKD46	λ Red recombinase plasmid	(77)
pCP20	Excision of antibiotic-resistant cassette	(84)

a) the relevant genotype of selective pore variant is described as amino acid mutations were made.
b) the relevant genotype of chimera variant is described as the amino acid position where the conjunctions of two proteins was made.

Supplemental Table S2. Primers used in this study

**Name**	**Sequence (5’ → 3’)**
**Deletion primers**	
TrkG-Del-F	ATCATAATATTGCAGCAAGGTGGTTATAATTGAAAGAATATTTAG ATATGATTCCGGGGATCCGTCGACC
TrkG-Del-R	TAAACTCACAATTATAAATCAGCCATATATTAGGAGCGCCAAAAA AAACCGTGTAGGCTGGAGCTGCTTC
TrkH-Del-F	GAAGAATAATCCCCACTTCGTTTGCAGAACTAAGGAAGCGGCAG AGATGATTCCGGGGATCCGTCGAC
TrkH-Del(Cm)-50F	GAAGAATAATCCCCACTTCGTTTGCAGAACTAAGGAAGCGGCAG AGATGCATATGAATATCCTCCTTAG
TrkH-Del-R	AAGAATTAATGTTTTCACGTATTACTCCATCATTCACGCCAGAAAG TCGGTGTAGGCTGGAGCTGCTTC
Kup-Del-F	AAGCACACATTTCATATTTCAACGAAAGACTAGTCTATGATTCCGG GGATCCGTCGAC
Kup-Del-R	GAAAGGAGGCGTCTGGCGTTAGATTTCGACCTGAGTACCTGTAGG CTGGAGCTGCTTC
KdpA-Del(Cm)-50F	TTACTGGGTTATCTGGTTTATGCCCTGATCAATGCGGAGGCGTTCT GATGCATATGAATATCCTCCTTAG
KdpA-Del-50R	GACTCATATTCAGTGCTCACTCAATATCATCAGGAGAGATATTCCG CCACTGTAGGCTGGAGCTGCTTC
NhaB-Del(Cm)-50F	TAAACATAAAAACATCATTGATTATTTGTAAGGTAAGGGAAACCA TCATGCATATGAATATCCTCCTTAG
NhaB-Del-50R	CCGGGCATTTATATGCCCGGTAAGTTGTTATCAAAGCGTTGCTATC CAGCTGTAGGCTGGAGCTGCTTC
**Cloning primers (wild-type)**	
pPAB404-BamHI-TrkG-F	CGGTACCCGGGGATCCATGAATACATCTCATGTA
pPAB404-PstI-TrkG-R	ATATCGCCCTGAGGCTGCAGTTAGGAGCGCCAAAAAAA
pPAB404-BamHI-TrkH-F	CGGTACCCGGGGATCCATGCATTTTCGCGCCATT
pPAB404-PstI-TrkH-R	ATATCGCCCTGAGGCTGCAGTCATTCACGCCAGAAAGT
**Cloning primers (selective pore variants)**	
TrkG-L223I-F	TTTCGATTGGCGGCTTCTCAACTCATAGC
TrkG-L223I-R	GTTGAGAAGCCGCCAATCGAAACTGTGGA
TrkH-I220L-F	TCGCTCTTGGTGGTTTCTCGACACATGAT
TrkH-I220L-R	GTCGAGAAACCACCAAGAGCGATAGTCGC
TrkG-D320T-N321A-L323F-F	TCACTACCGCCGGGTTTGCTACGCAGGAT
TrkG-D320T-N321A-L323F-R	GTAGCAAACCCGGCGGTAGTGAGCATGGA
TrkH T318D-A319N-F321L-F	CGACAGATAATGGTTTAACAACTGACAGC
TrkH T318D-A319N-F321L-R	GTTGTTAAACCATTATCTGTCGCCATCGA

Supplemental Table S2. (continued)

Name	Sequence (5' → 3')
TrkG-M438L-L440P-F	TTAATAACCTGGGGCCAGGTTTTGGGGCT
TrkG-M438L-L440P-R	AAACCTGGCCCCAGGTTATTAATACATGC
TrkH L436M-P438L- F	TGAATAATATGGGATTAGGGCTTGGCGTG
TrkH L436M-P438L-R	AGCCCTAATCCCATATTATTCAATGTCGC
**Cloning primers (chimera variants)**	
TrkG-S264-TrkH-G262-F	TTGTTATTAGCGGGCGTAGTCTGAAGGTT
TrkG-S264-TrkH-G262-R	AGACTACGCCCGCTAATAACAATATACCA
TrkH-S261-TrkG-R265-F	CACTGTTAAGTAGGAAAACGATAAAACCT
TrkH-S261-TrkG-R265-R	ATCGTTTTCCTACTTAACAGTGAAAAGTG
TrkG-G387-TrkH-N386-F	TAAATGTAGGAAATCGCGCACTGCCGGAA
TrkG-G387-TrkH-N386-R	AGTGCGCGATTTCCTACATTTACACTCAA
TrkH-P168-TrkG-F172-F	TGCCCGGCCCGTTTAAGGATGACAAACTC
TrkH-P168-TrkG-F172-R	TCATCCTTAAACGGGCCGGGCATTTCTGC
**Cloning primers (luciferase assay)**	
trkG_LUX_F	TCGTCTTCACCTCGATCGCCTACAAAGAACAGCGCGACAA
trkG_LUX_R	ACTAACTAGAGGATCATCTAAATATTCTTTCAATTATAAC
trkH_LUX_F	TCGTCTTCACCTCGATCAGGAACGAGGAACAGGGACAAAA
trkH_LUX_R	ACTAACTAGAGGATCCTCTGCCGCTTCCTTAGTTCTGCAA
kdpA_LUX_F	TCGTCTTCACCTCGAAAAGCGGGCGCGGTCTTTCCAGAAA
kdpA_LUX_R	ACTAACTAGAGGATCCAGAACGCCTCCGCATTGATCAGGG
**Universal primers**	
k1	CAGTCATAGCCGAATAGCCT
k2	CGGTGCCCTGAATGAACTGC
c1	ATACGCAAGGCGACAAGG
c2	TCTTCCGTCACAGGTAGG
**RT-qPCR primers**	
trkG-439bp-F	TTCTGGCGGTTGCTGTATTG
trkG-542bp-R	AGGCGGGGAGTGAGTTTG
trkH-370bp-F	CCTCACGCCATCCTCTTTTATC
trkH-448bp-R	GTATCGCAACCGCCAACAC
slp-339bp-F	CATCCTCGGCACCATTCAG
slp-487bp-R	CACCATAGCCGTAATCCCACA
hcaT-242bp-F	TTCTCTTTGCTGTCGCCTTC
hcaT-401bp-R	AGTCGCACTTTGCCGTAATC
cysG-648bp-F	TGAAGTGGTGCTGGTTGGTG
cysG-730bp-R	CATCTGCCTGCTGAATTTGTTG
idnT-1002bp-F	TTCGCCGTTATTGATGTGCT
idnT-1191bp-R	GCCAGGGTCGTTTACGTGTG

Supplemental Table S2. (continued)

Name	Sequence (5' → 3')
osmC- 175bp-F	TGTTTCTCAATGGCGCTTTC
osmC- 319bp-R	GCACCGCAACTTCACTCTTC
proV-189bp-F	CGGCTCGGGTAAATCCA
proV-272bp-R	TCCACACCATCAATCAGCAC
